# Resting state functional connectivity of the pain matrix and default mode network in irritable bowel syndrome: a graph theoretical analysis

**DOI:** 10.1038/s41598-020-67048-9

**Published:** 2020-07-03

**Authors:** Michiko Kano, Cecilia Grinsvall, Qian Ran, Patrick Dupont, Joe Morishita, Tomohiko Muratsubaki, Shunji Mugikura, Huynh Giao Ly, Hans Törnblom, Maria Ljungberg, Kei Takase, Magnus Simrén, Lukas Van Oudenhove, Shin Fukudo

**Affiliations:** 1 Sukawa clinic, Kirari health coop, Fukushima, Japan; 2 Behavioral Medicine, Graduate School of Medicine, Tohoku University, Sendai, Japan; 3 Psychosomatic Medicine, Tohoku University Hospital, Sendai, Japan; 4 Department of Internal Medicine & Clinical Nutrition, Institute of Medicine, Sahlgrenska Academy, University of Gothenburg, Gothenburg, Sweden; 5 Laboratory for Cognitive Neurology, KU Leuven, Leuven, Belgium; 6 Diagnostic Radiology, Tohoku University Hospital, Sendai, Japan; 7 Laboratory for Brain-Gut Axis Studies (LaBGAS), Translational Research Center for Gastrointestinal Disorders (TARGID), KU Leuven, Leuven, Belgium; 8 Department of Radiation Physics, Institute of Clinical Sciences, Sahlgrenska Academy, University of Gothenburg, Gothenburg, Sweden; 9 Department of Medical Physics and Biomedical Engineering, Diagnostic Imaging, Sahlgrenska University Hospital, MR Centre, Gothenburg, Sweden; 10 Cognitive and Affective Neuroscience Lab, Department of Psychological & Brain Sciences, Dartmouth College, Hanover, NH USA

**Keywords:** Chronic pain, Irritable bowel syndrome

## Abstract

Irritable bowel syndrome (IBS) is a functional disorder of brain-gut interactions. Differential brain responses to rectal distention between IBS and healthy controls (HCs) have been demonstrated, particularly in the pain matrix and the default mode network. This study aims to compare resting-state functional properties of these networks between IBS patients and HCs using graph analysis in two independent cohorts. We used a weighted graph analysis of the adjacency matrix based on partial correlations between time series in the different regions in each subject to determine subject specific graph measures. These graph measures were normalized by values obtained in equivalent random networks. We did not find any significant differences between IBS patients and controls in global normalized graph measures, hubs, or modularity structure of the pain matrix and the DMN in any of our two independent cohorts. Furthermore, we did not find consistent associations between these global network measures and IBS symptom severity or GI-specific anxiety but we found a significant difference in the relationship between measures of psychological distress (anxiety and/or depressive symptoms) and normalized characteristic path length. The responses of these networks to visceral stimulation rather than their organisation at rest may be primarily disturbed in IBS.

## Introduction

Irritable bowel syndrome (IBS) is a highly prevalent gastrointestinal disorder characterized by chronic recurrent abdominal pain associated with altered bowel habits in the absence of organic causes detected with routine medical examinations^1^. IBS is referred to as a functional gastrointestinal disorder (FGID) or, since the recent Rome IV process, a disorder of gut-brain interactions (DGBI)^1^. Around 11% of the population are affected by IBS worldwide, which causes a significant personal and societal burden globally^2^. The etiology and pathogenesis of IBS is poorly understood but likely multifactorial, as abnormalities of the gut microbiota, epithelial barrier function, and immune system function, as well as neuroendocrine mechanisms, all have been identified as (peripheral) biological mechanisms potentially contributing to IBS pathophysiology and symptom generation^3^. At the level of gastrointestinal function, visceral hypersensitivity and altered gut motility have been identified in subgroups of IBS patients, and these alterations have been shown to be associated with symptom severity^4,5^. However, frequent psychiatric and psychological co-morbidities and their ability to interfere with processing and modulation of afferent gut-brain signals as well as gastrointestinal (GI) motor and barrier function through the gut-brain axis points towards an important role for central nervous system processes in IBS^6,7^. Therefore, IBS has been explicitly conceptualized as a disorder of altered brain-gut interactions in the Rome IV consensus^8^, and a biopsychosocial model has been adopted to understand symptoms of IBS^6^.

Functional brain imaging studies have indeed demonstrated differential responses to controlled rectal distension between IBS and healthy controls in a “(visceral) pain neuromatrix” (i.e. the network of pain-responsive regions) consisting of functionally distinct but highly interacting subnetworks, each of which contribute to the experience of and response to (visceral) pain. The regions of the pain matrix can be subdivided into: (1) sensorimotor areas (e.g. thalamus, posterior insula, and basal ganglia), (2) salience areas (e.g. anterior midcingulate cortex (aMCC), anterior insula, and amygadala), (3) emotional arousal areas (e.g. amygdala, hippocampus, pregenual and subgenual anterior cingulate cortex [pgACC, sgACC], medial prefrontal cortex [mPFC], (4) descending pain modulation and central autonomic network (e.g. hypothalamus, periaqueductal gray [PAG], Locus coeruleus complex [LCC], amygdala, anterior insula, aMCC, and mPFC) (5) central executive network (e.g. dorsolateral prefrontal cortex [dlPFC] and posterior parietal cortex [PCC])^9–13^. These pain-responsive networks dynamically interact with the default-mode network (DMN), which is active when attention is not directed to a specific exteroceptive or interoceptive stimulus. When visceral pain stimuli are processed in the sensorimotor network and activate the salience network, activity is shifted away from the default network towards the central-executive network which allocates cognitive resources to the salient interoceptive stimulus and engages the emotional-arousal and central autonomic network resulting in affective activation and appropriate response selection^13^.

Despite an increasing use of resting-state functional magnetic resonance imaging (rs-fMRI) in health and disease^14–16^, including other chronic functional pain disorders such as fibromyalgia^17,18^, rs-fMRI studies in IBS are sparse compared to task-based fMRI studies during which the brain response to pain is measured. Furthermore, the existing studies suffer from a number of limitations including small and heterogeneous samples, lack of stringent multiple testing control, and use of highly variable methods, all of which render comparison of studies difficult and may account for discrepant findings. That said, differences in resting-state brain function and/or connectivity have been described in IBS versus healthy controls, in the DMN as well as in key pain neuromatrix regions^16,19–31^.

In addition to functional connectivity between specific regions, the functional properties of the entire brain network can be analysed using a graph theoretical approach. Graph theory provides a theoretical framework in which the topology of complex networks can be examined, and can reveal important information about both the local and global organization of functional brain networks^32^. However, this approach has only rarely been used to analyse rs-fMRI data in IBS^19,23^.

Therefore, the aim of this study is to compare resting-state functional properties of a network consisting of pain matrix and default mode network regions between IBS patients and healthy controls using graph analysis in two independent cohorts analysed using the same pipeline. Further, we aimed to explore the relationships between these measures and measures of GI and psychological symptom severity. Based on the above-mentioned earlier task-based and rs-fMRI studies in IBS and healthy controls, we expected significant global differences in these functional properties between IBS patients and controls, as well as significant relationships between these properties and measures of somatic and psychological symptom severity.

## Results

### Descriptive statistics

#### Sendai cohort

No differences in age or sex distribution were found between IBS patients (n = 30) and HC (n = 29). IBS patients scored significantly higher than HC on IBS symptom severity and GI-specific anxiety, but not on trait anxiety or depressive symptoms (Table 1).

**Table 1:** Descriptive characteristics of both cohorts.

	healthy controls	IBS patients	p-value
**Sendai cohort**			
n	29	30	
age	22 [20–23]	21 [20–23]	0.56
sex (m/f)	15/14 (52/48%)	13/17 (43/57%)	0.52°
*IBS-SSS GI symptoms*	*41* [12–80]	*179.5 [139–203]*	*<0.0001*
STAI trait anxiety	36 [33–40.5]	38.5 [31.5–45]	0.38
SDS depressive symptoms	35.5 [32.5–38]	35.5 [31–43.5]	0.73
*VSI GI-specific anxiety*	*1 [0–5]*	*24.5 [13.5–33]*	*<0.0001*
**Gothenburg cohort**			
n	29	62	
age	29 [26–33]	31 [26–39]	0.29
sex (m/f)	10/19 (34/66%)	17/45 (27/73%)	0.49°
*IBS-SSS GI symptoms*	*12* [4–29]	*295 [197–358]*	*<0.0001*
*IBS-SSS extracolonic symptoms*	*33.5 [15.5–52.5]*	*155.25 [100.5–223.0]*	*<0.0001*
*HADS anxiety symptoms*	*4* [1–5]	*9* [6–12]	*<0.0001*
*HADS depressive symptoms*	*1 [0–2]*	*5* [3–7]	*<0.0001*
*VSI GI-specific anxiety*	*1 [0–2]*	*39* [24–51]	*<0.0001*

Values are median [25th percentile-75th percentile] or n (%); p-values are from Kruskal-Wallis non-parametric one-way ANOVAs, except for ° from Pearson χ² test; italic indicates significant group differences.

IBS, irritable bowel syndrome; IBS-SSS, IBS severity scoring system; STAI, Spielberger State-Trait Anxiety Inventory; SDS, Zung Self-rating Depression Scale; HADS, hospital anxiety & depression scale; VSI, visceral sensitivity index.

#### Gothenburg cohort

No differences in age or sex distribution were found between IBS patients (n = 62) and HC (n = 29). IBS patients scored significantly higher on IBS symptoms, extra-colonic symptoms, anxiety symptoms (both general and GI-specific) and depressive symptoms compared to HC (Table 1).

It should be noted that the Sendai cohort consists of less severe IBS patients compared to the Gothenburg cohort, as evident from the lower IBS-SSS and VSI scores, as well as from the fact that trait anxiety and depressive symptom ratings were low and not significantly different from HC in the Sendai cohort.

### Global graph measures in IBS patients compared to healthy controls

**Figure 1:**
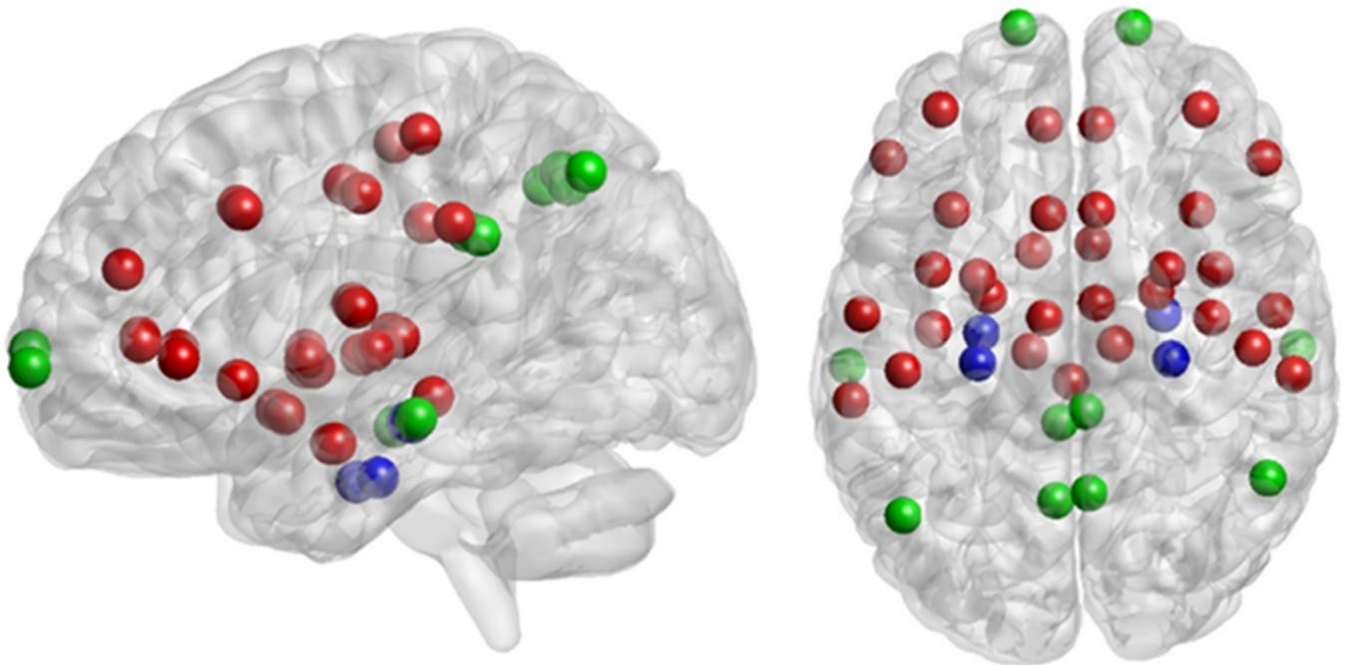
Visualisation of all nodes of the network used in the default mode network (green + blue) and the “pain” network (red + blue). Nodes are projected on a sagittal (left) or a top view (right). Note that the position of the node corresponds to the center of mass of the region we used but that it is not representing the actual region outline. Visualisation is performed using the BrainNet Viewer^67^.

The nodes of the network are demonstrated in Table 2 and Fig. 1.

**Table 2:** List of volumes of interest representing the nodes of the network.

Short name	Full name	atlas
mPFC	medial prefrontal cortex	Destrieux
dlPFC	dorsolateral prefrontal cortex	Brodmann
vlPFC	ventrolateral prefrontal cortex	Destrieux
pACC	perigenual anterior cingulate cortex	Destrieux
sACC	subgenual anterior cingulate cortex	Destrieux
aMCC	anterior middle cingulate cortex	Destrieux
pMCC	posterior middle cingulate cortex	Destrieux
PCC	posterior cingulate cortex	Destrieux
insula anterior	anterior insula	*Larsson et al. 2012*
insula mid	middle insula	*Larsson et al. 2012*
insula posterior	posterior insula	*Larsson et al. 2012*
amygdala	amygdala	AAL
parahippo	parahippocampal gyrus	Destrieux
hippocampus	hippocampus	Destrieux
SII	secondary somatosensory cortex	Destrieux
SI	primary somatosensory cortex	Destrieux
LTC	lateral temporal cortex	Destrieux
IPL	inferior parietal lobule	Destrieux
precuneus	precuneus	Destrieux
Angular	Angular gyrus	Destrieux
putamen	putamen	AAL
thalamus	thalamus	AAL
PAG	peri-aqueductal gray	*sphere around 0,−28,−8*

#### Sendai cohort

**Figure 2:**
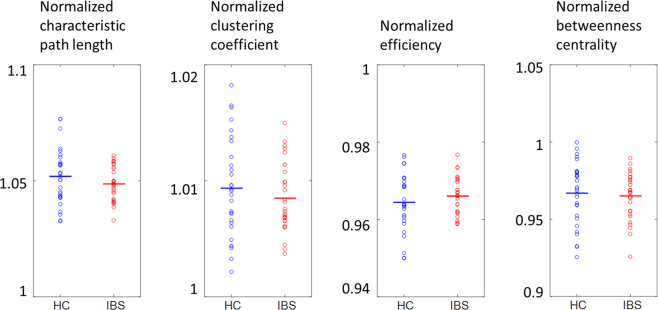
Distribution of the values for the normalized characteristic path length, clustering coefficient, global efficiency and betweenness centrality for HC or IBS for the Sendai cohort. None of the differences was significant (all p > 0.2). HC: healthy controls, IBS: irritable bowel syndrome.

No significant differences in global graph measures between IBS patients and HC were found (all p-values > 0.2) (Table 3, Fig. 2).

**Table 3:** Normalized global graph measures in IBS patients compared to healthy controls.

Normalized graph measure	healthy controls	IBS patients	p-value
***Sendai cohort***			
clustering coefficient	1.0094 ± 0.0007	1.0085 ± 0.0007	0.37
efficiency	0.9644 ± 0.0012	0.9660 ± 0.0012	0.33
betweenness centrality	0.9668 ± 0.0033	0.9651 ± 0.0032	0.70
characteristic path length	1.0519 ± 0.0019	1.0487 ± 0.0018	0.22
**Gothenburg cohort**			
clustering coefficient	1.0077 ± 0.0006	1.0079 ± 0.0004	0.83
efficiency	0.9701 ± 0.0013	0.9695 ± 0.0009	0.73
betweenness centrality	0.9726 ± 0.0035	0.9700 ± 0.0023	0.55
characteristic path length	1.0432 ± 0.0021	1.0442 ± 0.0015	0.70

Values are averages (±SEM) of normalized graph measures; p-values from independent samples t-tests.

IBS, irritable bowel syndrome.

#### Gothenburg cohort

**Figure 3:**
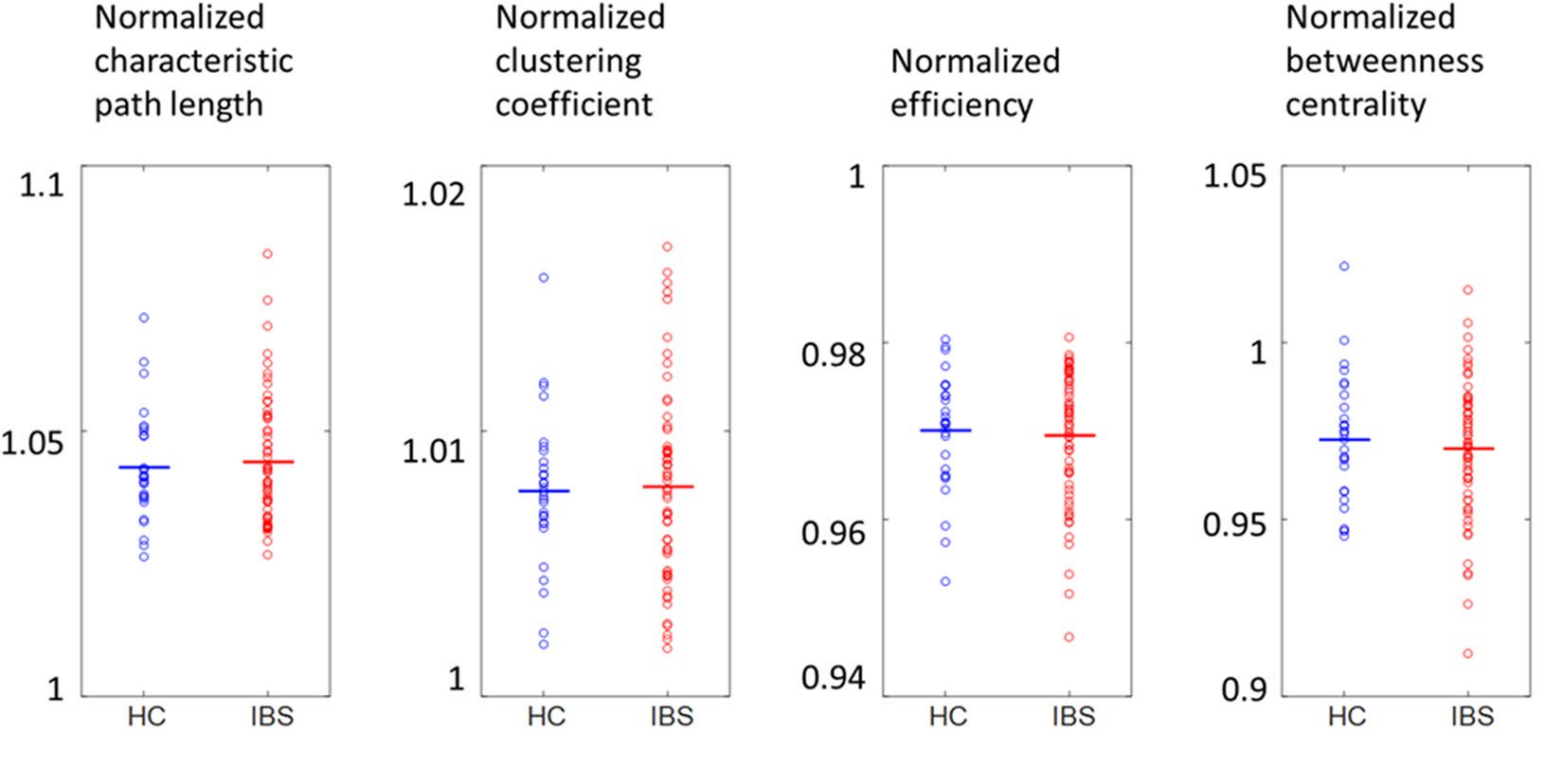
Distribution of the values for the normalized characteristic path length, clustering coefficient, global efficiency and betweenness centrality for HC or IBS for the Gothenburg cohort. None of the differences was significant (all p > 0.5). HC: healthy controls, IBS: irritable bowel syndrome.

No significant differences in global graph measures between IBS patients and HC were found (all p-values > 0.5) (Table 3, Fig. 3).

### Hubs in IBS patients compared to healthy controls

#### Sendai cohort

The nodes identified as hubs (versus equivalent random networks) in healthy controls and IBS patients are summarized in Table 4. The right angular gyrus is the only common hub, while dlPFC is specific for HC, and right precuneus, left angular gyrus, and left SII are specific to IBS patients. Left SII remained significantly more likely to be a hub in IBS patients compared to healthy controls after FDR correction (Table 5).

**Table 4:** Overview of hubs in IBS patients and healthy controls.

healthy controls	IBS patients
**Sendai cohort**	
right angular gyrus	right angular gyrus
right dlPFC	left angular gyrus
	left SII
	right precuneus
**Gothenburg cohort**	
right precuneus	right precuneus
left precuneus	left angular gyrus
	right angular gyrus
	left IPL
	right IPL
	left SI
	right SI
	left dlPFC
	right dlPFC
	left mPFC

IBS, irritable bowel syndrome; PCC, posterior cingulate cortex; dlPFC, dorsolateral prefrontal cortex; IPL, inferior parietal lobule; SI/II, primary/secondary somatosensory cortex; mPFC, medial prefrontal cortex.

**Table 5:** Significant differences in probability to be a hub between IBS patients and healthy controls.

node	% hub in healthy controls	% hub in IBS patients	p-value (uncorrected)
**Sendai cohort**			
left anterior insula	28	10	0.038
left middle insula	3	20	0.020
left SI	21	40	0.049
left SII	7	57	<0.0001*
right PCC	45	20	0.018
right putamen	21	3	0.017
**Gothenburg cohort**			
left angular gyrus	21	48	0.002
left middle insula	28	11	0.038
left mPFC	21	39	0.032
left pACC	41	19	0.017
right hippocampus	17	3	0.029
right LTC	17	32	0.050
right sACC	17	2	0.015

*Significant after FDR correction for multiple testing.

IBS, irritable bowel syndrome; LTC, lateral temporal cortex; PCC, posterior cingulate cortex; SII, secondary somatosensory cortex; mPFC, medial prefrontal cortex; pACC, pregenual anterior cingulate cortex; sACC, subgenual anterior cingulate cortex.

#### Gothenburg cohort

The nodes identified as hubs (versus equivalent random networks) in healthy controls and IBS patients are summarized in Table 4. The right precuneus is the only common hub, while left precuneus is specific for HC, and bilateral angular gyrus, bilateral IPL, bilateral SI, bilateral dlPFC, and left mPFC are specific for IBS patients. However, after FDR correction, no significant differences in probality to be a hub were found between HC and IBS (Table 5).

### Modularity structure in IBS patients compared to healthy controls

#### Sendai cohort

**Figure 4:**
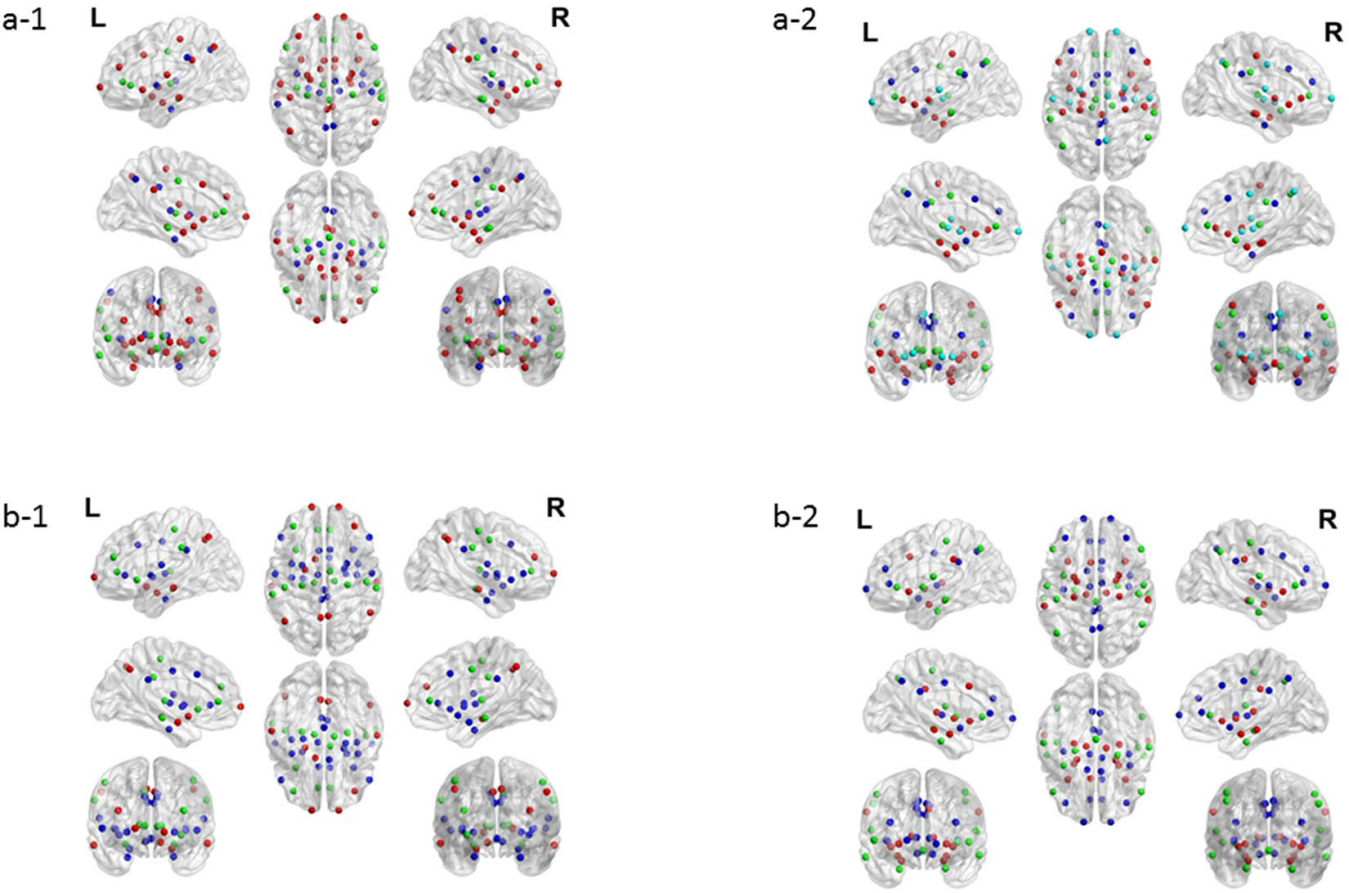
Visualization of the modularity structure in healthy controls (left panels a-1 and b-1) and IBS patients (right panels a-2 and b-2) for the Sendai cohort (top panels a-1 and a-2) and the Gothenburg cohort (bottom panels b-1 and b-2). Regions with the same color belong to the same module. Visualisation is performed using the BrainNet Viewer^67^.

The modularity structure in IBS patients and HC is summarized in Table 6 and Fig. 4a. Three and four modules were identified in HC and IBS patients, respectively.

**Table 6:** Modularity structure in IBS patients and healthy controls.

**Sendai cohort**			
**HC**			
L/R amygdala	L/R hippocampus	L/R middle insula	
L/R angular gyrus	L/R pACC	L/R thalamus	
L/R anterior insula	L/R vlPFC	L/R precuneus	
L LTC	PAG	L IPL	
L/R PCC	L posterior insula	L parahippocampal gyrus	
L/R putamen	L pMCC	R posterior insula	
L SI	R IPL	R SI	
L SII	R LTC	R pMCC	
L/R aMCC	R SII		
L/R dlPFC			
L/R mPFC			
R parahippocampal gyrus			
L/R sACC			
**IBS**			
L/R amygdala	L/R angular gyrus	L/R PCC	L/R posterior insula
L/R hippocampus	L/R IPL	L/R aMCC	L/R putamen
L/R anterior insula	L/R thalamus	L/R dlPFC	L/R SII
L/R middle insula	L/R pACC	L/R precuneus	L/R mPFC
L/R vlPFC	L LTC	L sACC	R pMCC
L parahippocampal gyrus	L pMCC	R parahippocampal gyrus	
L/R SI	R sACC		
PAG			
R LTC			
**Gothenburg cohort**			
**HC**			
L/R angular gyrus	L/R hippocampus	L/R middle insula	
L/R LTC	L/R IPL	L/R posterior insula	
L/R mPFC	L/R thalamus	L/R PCC	
L/R precuneus	L/R SI	L/R putamen	
L amygdala	L/R pACC	L/R SII	
L sACC	L anterior insula	L/R aMCC	
R dlPFC	L dlPFC	L/R parahippocampal gyrus	
	R pMCC	L/R vlPFC	
		L pMCC	
		PAG	
		R amygdala	
		R anterior insula	
		R sACC	
**IBS**			
L/R amygdala	L/R angular gyrus	L/R posterior insula	
L/R hippocampus	L/R LTC	L/R PCC	
L/R anterior insula	L/R SI	L/R aMCC	
L/R IPL	L/R SII	L/R dlPFC	
L/R putamen	L/R parahippocampal gyrus	L/R mPFC	
L/R thalamus	L/R vlPFC	L/R pACC	
	L middle insula	L/R pMCC	
	PAG	L/R precuneus	
		L/R sACC	
		R middle insula	

IBS, irritable bowel syndrome; L, left; R, right; IPL, inferior parietal lobule; LTC, lateral temporal cortex; PCC, posterior cingulate cortex; SI/SII, primary/secondary somatosensory cortex; aMCC, anterior midcingulate cortex; dlPFC, dorsolateral prefrontal cortex; mPFC, medial prefrontal cortex; pACC, pregenual anterior cingulate cortex; pMCC, posterior midcingulate cortex; sACC, subgenual anterior cingulate cortex; vlPFC, ventrolateral prefrontal cortex; PAG, periaqueductal grey matter.

Significant differences in modularity structure between IBS patients and HC at the uncorrected level (pairwise differences in probability to belong to the same module) are summarized in Supplementary Table S1. None of these differences survived an FDR correction for multiple testing.

#### Gothenburg cohort

The modularity structure in IBS patients and HC is summarized in Table 6 and Fig. 4b. In both groups, three modules were identified.

Significant differences in modularity structure between IBS patients and HC at the uncorrected level (pairwise differences in probability to belong to the same module) are summarized in Supplementary Table S1. None of these differences survived an FDR correction for multiple testing.

### Relationship between graph measures and symptom severity measures

#### IBS symptom severity and GI-specific anxiety (IBS patients only)

Sendai cohort. An overview of correlations between IBS-SSS scores (colonic), VSI score, and global graph measures is given in Table 7. For IBS symptoms, a significant positive correlation with a medium effect size was found with normalized betweenness centrality, whereas for GI-specific anxiety, significant positive and negative correlations of medium magnitude were found with normalized betweenness centrality and normalized characteristic path length, respectively. All these correlations remained significant after FDR correction for multiple testing.

**Table 7:** Correlations between GI/somatic symptom severity, GI-specific anxiety, and normalized global graph measures.

Sendai cohort			
Normalized graph measure	IBS-SSS score colonic		VSI score
clustering coefficient	0.26		0.16
betweenness centrality	0.39*^		0.45*^
characteristic path length	0.04		−0.45*^
**Gothenburg cohort**			
**Normalized graph measure**	**IBS-SSS score colonic**	**IBS-SSS score extra-colonic**	**VSI score**
clustering coefficient	−0.20	−0.26*	−0.04
betweenness centrality	0.01	0.14	0.04
characteristic path length	−0.24°	−0.24°	−0.12

Values are Spearman’s ρ; °p < 0.07 (uncorrected); *p < 0.05 (uncorrected); ^p < 0.05 (FDR corrected).

IBS, irritable bowel syndrome; IBS-SSS, IBS severity scoring system; VSI, visceral sensitivity index.

Gothenburg cohort. An overview of correlations between IBS-SSS scores (colonic and extra-colonic), VSI score, and normalized global graph measures is given in Table 7.

#### Co-morbid anxiety and depressive symptoms

Sendai cohort. The results of the ANCOVA analyses are summarized in Table 8.

**Table 8:** Results of ANCOVA analyses testing the relationship between levels of psychological distress and normalized graph measures.

	main effect group	main effect psychological distress	group-by-psychological distress interaction
**Sendai cohort**			
***trait anxiety***			
Normalized clustering coefficient	2.87	2.65	1.13
Normalized betweenness centrality	0.01	0.03	0.10
Normalized characteristic path length	4.11*	1.86	2.38
***depressive symptoms***			
Normalized clustering coefficient	2.63	4.44*	3.63°
Normalized betweenness centrality	0.00	0.19	0.74
Normalized characteristic path length	4.71*	6.78*	6.95*
**Gothenburg cohort**			
Normalized clustering coefficient	0.01	0.02	2.10
Normalized betweenness centrality	0.86	0.85	0.01
Normalized characteristic path length	0.28	0.25	4.20*

Values are F-statistics; °p < 0.07, *p < 0.05.

For normalized characteristic path length, a significant group-by-depressive score interaction effect was found, driven by a significantly positive relationship between level of depressive symptoms and normalized characteristic path length in HC (r = 0.48, p = 0.009), which was absent in IBS patients (r = −0.006, p = 0.97). A similar trend was found for normalized clustering coefficient.

Gothenburg cohort. The results of the ANCOVA analyses are summarized in Table 8.

Like in the Sendai cohort, there was a significant group-by- psychological distress interaction effect for characteristic path length, driven by a positive and negative relationship between level of psychological distress and characteristic path length in HC (r = 0.30, p = 0.11) and IBS patients (r = −0.20, p = 0.13), respectively.

Whole brain analysis using the Shen 50 atlas. Additionally, we performed a whole-brain analysis using the Shen 50 atlas^33^, which is a whole brain parcellation based on resting state fMRI in which 50 parcels per hemisphere were calculated. We found no significant differences in graph measures nor in their association with psychological scores between HC and IBS groups (Supplementary Material Tables S2–S7).

## Discussion

The current study aimed to characterize functional properties of a brain network consisting of DMN and pain-responsive areas by graph analysis in patients with IBS and HCs in two independent cohorts from Sendai and Gothenburg. There were no significant differences between IBS patients and HCs on any of the global graph measures. The hubs and modularity structures could not be replicated between the Sendai and Gothenburg cohorts. This was also the case for the correlation between symptom severity measures and graph measures. There was a significant interaction between depression score and group for characteristic path length, driven by a significant positive relationship in HCs in the Sendai cohort. Similarly, there was a significant interaction between psychological distress and characteristic path length, driven by a positive relationship in HCs and a negative relationship in Gothenburg IBS group.

### Methodological adequacy to support the non-significant results

When looking at the graph analysis as a whole, we found a lack of significance and rather high variability in the difference between HCs and patients with IBS between the Sendai and Gothenburg cohorts. This can be at least partly explained by different features of the two cohorts and/or technical differences (see limitations paragraph below). To shed more light on this remarkable and unexpected variability between both cohorts, even for HCs, we performed additional analyses. *First*, we investigated test-retest reliability of the graph analysis using the exactly same methods on a publicly available dataset scanning twice within 6 months. The low test-retest variability of graph measures between the two scans indicates sufficient reproducibility (see Supplementary Material). However, identification of hubs and the modular structure was not very reproducible (see Supplementary Material), implying that this may at least partially account for the variability in results between the Sendai and Gothenburg cohorts. *Second*, comparison of graph measures between HCs from the Sendai and the Gothenburg cohorts showed significant differences in normalized efficiency, even when taking into account multiple testing correction, which prevented us from pooling both cohorts in the same analysis (see Supplementary Material). These results indicate that the global graph measures we used in the current analysis have good test-retest reliability and good sensitivity to detect differences, even between HCs from different cohorts, thereby increasing confidence in our negative results when comparing IBS patients and HCs within each cohort.

We also tried to replicate the previous paper which performed graph analysis on rsfMRI data in healthy controls and IBS patients, and found a significant decrease in global efficiency in patients with IBS compared with HCs^23^. As there are a number of methodological differences between that study and ours, we re-analyzed our data according to their method except for the node selection (see Supplementary Material). We could not find a significant difference between IBS patients and HCs in global efficiency in none of our two cohorts (see Supplementary Material).

Furthermore, we conducted another analysis using nodes covering the whole brain and used the same techniques we used in the original study to investigate whether the negative results could be due to the node selection limited to the pain matrix and DMN (see Supplementary Material). Using this approach, we found no significant differences in graph measures, nor in their association with psychological scores between IBS and HC groups (see Supplementary Material), thereby confirming our primary analysis in a more limited set of regions. Thus, the global organization assessed by graph analysis may not be different between IBS and HCs in a whole-brain network nor in a more limited network consisting of the pain matrix and DMN.

### Differential associations between psychological distress and normalized characteristic path length in IBS patients and HCs

A significant group-by-depressive symptoms interaction was found for normalized characteristic path length in the Sendai cohort, whereas a similar significant group-by-psychological distress interaction effect was found in the Gothenburg cohort. The interactions were driven by a positive relationship between levels of depressive symptoms/psychological distress in HC in both cohorts, and negative or no relationship in IBS patients in the Gothenburg and Sendai cohorts, respectively. This is the only finding observed consistently across both cohorts in the current study. However, we could not find this association when looking at graph measures derived from a whole-brain network. In their abovementioned paper, Qi *et al*. reported that the difference of DMN global efficiency observed between HCs and IBS patients was partially accounted for by differences in anxiety and depression. However, they did not report on any (differential) associations between levels of anxiety or depressive symptoms and global graph measures, rendering comparison with our results impossible^23^. Nonetheless, our results indicate that levels of psychological distress (even if subsyndromal) may be associated with global characteristic path length in HCs but not IBS patients. Global characteristic path length is a measure of the cost of information transfer within a network. There have been reports indicating differences in global characteristic path length in patients with major depression in different networks including the DMN, but results have been inconsistent in that both increases and decreases have been found^34–37^. The interaction effect in the current study indicates a positive association between psychological distress scores and global functional organisation of the pain matrix and DMN in HCs, which may not exist in a pathological condition such as IBS. Differential associations between psychological features and brain function metrics in IBS patients and HCs have been reported before, including in our own previous work where we demonstrated significant associations between alexithymia scores and brain responses to rectal pain in HCs but not in IBS patients^38^.

### Strengths and limitations of this study

The current study has a number of strengths. First, we checked the test-retest reliability of graph measures in another dataset using the same processing pipeline within the pain matrix and DMN network. Second, we adopted exactly the same preprocessing and graph analysis pipeline in two independent cohorts, whereas all the previous studies were performed in a sample recruited from a single center. Together with the rather small samples used, this may impair generalizability of findings as IBS cohorts, like cohorts of other symptom-based diagnoses, are heterogeneous in terms of pathophysiology, e.g. some patients may have primarily gut-oriented pathophysiology while others may be primarily characterized by central dysfunctions^39^. In line with such heterogeneity, the current study showed important variability between the two independent cohorts, indicating that caution is needed to generalize results from a single-center cohort or a study with a small number of participants to the IBS population as a whole. Considering the heterogeneity of IBS patients, we may need larger cohorts and more extensive phenotyping, as well as robust methods, to study whether subgroups of IBS patients may be characterized by alterations in resting state function of the pain matrix and DMN.

The current study also has several limitations. *First*, there are important differences between both our cohorts in terms of age, race and cultural background. Together with differences in scanner and acquisition protocol, this may have accounted for the significant differences between the healthy controls from both sites described above, which prevented us from pooling both cohorts. However, this at the same time increases the generalizability of our negative findings when it comes to differences between IBS patients and controls, which may be considered a strength. Second, IBS patients differed between both cohorts in terms of severity of IBS and psychological symptoms, with the Gothenburg cohort being more severely affected. *Third*, although this is the largest rs-fMRI study in IBS versus controls published to date, sample sizes remain rather small compared to similar studies in other fields such as neurodegenerative disorders. Larger sample sizes may be reached by pooling data from different centers, but as mentioned above, our data shows that this may not be without problems, at least for a graph theoretical analysis of resting state fMRI data.

### Future work

One reason that made it difficult to compare our results with previous studies, is the large variability in analytical approaches (e.g. methods to assess functional connectivity, selection of the nodes of a network, targets such as microbiota composition and/or sensorimotor function^19,25^ for correlating with functional connectivity or graph measures, or psychological functioning^29^). In addition to methodological variations, there are a number of limitations in all previous studies, including small sample sizes, lack of stringent correction for multiple testing, absence of validation cohorts, and different analyses of the same small sample. Also, the heterogeneity of IBS needs to be properly addressed. Therefore, not only a large cohort is warranted but also an extensive but reasonable phenotyping is required. Although altered responses in the pain matrix and DMN networks to visceral stimulation (or its anticipation) in patients with IBS were repeatedly reported, the present study suggests that the organisation in the network at rest is not disturbed in IBS. A study to perform both functional brain imaging to visceral stimulation and resting state imaging in the same large cohort may be able to validate the finding.

## Conclusion

As a conclusion, we assessed the resting-state global functional organization of the pain matrix and the DMN between IBS patients and healthy controls in two independent cohorts by graph analysis. Global graph measures were not different between HCs and IBS patients in any of both cohorts. The association between psychological distress and normalized characteristic path length was different between HCs and IBS, a finding that was observed in the two cohorts. However, other results were considerably variable between the two cohorts. These results may indicate that the response of these networks to visceral stimulation rather than their organization at rest are primarily disturbed in IBS. This may have implications for future studies, particularly when aiming to identify brain-based diagnostic, prognostic, or therapeutic biomarkers for the disorder.

## Methods

### Subjects

#### Sendai cohort

In the first study, 35 IBS patients (16 males; mean age 22.4 ± 3.6 years) diagnosed according to the ROME III criteria^40^ and 33 healthy controls (HC) (18 males; mean age 22.2 ± 2.8 years) participated in a resting state fMRI experiment performed in Sendai (Japan). All patients with IBS belonged to non-constipated subtypes (31 diarrhea-predominant subtype [IBS-D] and 4 mixed subtype [IBS-M]). IBS subjects were recruited by advertisement and from the outpatient clinic in Tohoku University Hospital between 2011 and 2014. Each subject underwent a medical history review to exclude individuals with organic diseases a priori, as well as a physical examination by a gastroenterology & psychosomatic medicine specialist (MK). The exclusion criteria were a history of any mental and organic diseases including abdominal surgery and endocrine disease, as well as metal implants and claustrophobia for MRI scanning. Some participants in this study partially overlap with our previously published task-based functional MRI studies, without any overlap in research questions^10,41,42^,^38^.

Both HCs and IBS patients were asked to complete the following validated questionnaires in Japanese: the IBS Severity Index (IBS-SI in Japanese, corresponding to the IBS-Severity Scoring System (IBS-SSS) in English)^43^, the Zung Self-rating Depression Scale (SDS)^44,45^, the trait scale of the State-Trait Anxiety Inventory (STAI-T)^46,47^, and the Visceral Sensitivity Index (VSI) as a measure of GI-specific anxiety^48,49^.

Out of the 68 included subjects, 9 were excluded (5 IBS patients, 4 HC) due to excessive head movement during the rs-fMRI run (see below for criteria). The final analyzed sample for the Sendai cohort therefore consisted of 30 IBS patients and 29 healthy controls (see Table 1 for demographic information).

#### Gothenburg cohort

In a second independent study, 77 IBS patients (22 males; mean age 32.9 ± 1.2 years) and 36 HC (16 males; mean age 32.1 ± 1.6 years) participated in a similar rs-fMRI scanning session performed in Gothenburg (Sweden) as part of a larger longitudinal study on the pathophysiology of IBS. The group of IBS patients were diagnosed according to the Rome III criteria and they consisted of 13 patients with predominant constipation (IBS-C), 36 patients with predominant diarrhea (IBS-D), 9 patients with mixed symptoms (IBS-M), and 19 which were unclassified(IBS-U). For this purpose, IBS patients were recruited at the gastroenterology outpatient clinic specializing in functional GI disorders at Sahlgrenska University Hospital in Gothenburg between 2011 and 2014. IBS patients (18–65 years) came through self-referral or were referred by other physicians, mostly primary care doctors. The IBS diagnosis was based on clinical presentation, fulfilment of the Rome III criteria for IBS, and additional investigations if considered necessary by the gastroenterologist (HT or MS). Exclusion criteria included abnormal results on standard screening laboratory tests, severe psychiatric (presence of a psychiatric disease that dominated the clinical picture, i.e. that was the predominant complaint of the patient), systemic or other GI diseases, history of drug or alcohol abuse, and the inability to reliably respond to questionnaires in Swedish. Healthy controls were recruited by use of advertisement and checked by interview and questionnaire to exclude chronic diseases and any current GI symptoms. This study population has been used in a previous study on the relationship between brain functional connectivity measures and peripheral aspects of GI function, including microbiota composition and sensorimotor function, without any overlap with the previous research question or analysis^19^.

Both IBS patients and healthy controls completed the following validated questionnaires in Swedish: the IBS-SSS and VSI (identical to the Sendai cohort), and the Hospital Anxiety and Depression Scale (HADS), of which the total score (anxiety and depression subscales combined) was used as a measure of psychological distress as per recent recommendations^50^.

Out of the 113 included subjects, 16 were excluded (11 IBS patients, 5 HC) due to: (1) pathologies found on structural MRI scan (2 IBS patients); (2) exclusion criteria revealed after scanning (GI symptoms/disease, medication intake, previous pelvic surgery, 5 HC); (3) problems with data acquisition (3 IBS patients); (4) invalid IBS diagnosis due to discovery of organic cause (2 IBS patients); and (5) excessive head movement during the rs-fMRI run (see below for criteria) (8 IBS patients, 2 HC). The final analyzed sample for the Gothenburg cohort therefore consisted of 62 IBS patients and 29 healthy controls (Table 1).

#### Ethics

Subjects were given a description of the study protocol, and they provided written informed consent for participation. This study was approved by the Ethics Committees of Tohoku University School of Medicine (study 1) and Regional Ethical Review Board in Gothenburg (study 2). Both studies were conducted in accordance with the Declaration of Helsinki.

### MRI acquisition

Image acquisition was performed on a 3 T MRI. In Sendai, a structural MRI and a resting state fMRI were acquired on a Siemens Magnetom equipped with a 32-channel head coil. The structural scan was acquired using a T1-weighted magnetization prepared rapid acquisition gradient echo sequence (MPRAGE) to obtain 160 sagittal slices with a voxel size of 1.0 × 1.0 mm^2^ and a slice thickness of 1.1 mm using an MRPAGE sequence (TR = 2800 ms, TE = 2.98 ms, IT = 900 ms, flip angle 9°). The rs-fMRI scan was acquired as 250 volumes (excluding dummy scans) each containing 32 transaxial slices with an echo-planar imaging sequence (voxel size 3.4 × 3.4 × 4.0 mm^3^, TR = 1.8 s, TE = 30 ms, flip angle 90°, TA = 7min30s). During the rs-fMRI the subjects closed their eyes but were not allowed to sleep. In Gothenburg, a structural MRI and a resting state fMRI were acquired on a Philips Achieva equipped with an 8-channel phase-array head coil. The structural scan was acquired as 176 transverse slices with a voxel size of 1.0 × 1.0 mm^2^ and a slice thickness of 1.0 mm using a turbo field echo (TFE) sequence (TR = 2200 ms, TE = 3.2 ms, IT = 837 ms, flip angle 9°). The rs-fMRI scan was acquired as 300 volumes (excluding dummy scans), but only the first 250 volumes were used in order to perform analysis on the same amount of volumes as in the Sendai rs-fMRI data, with an echo-planar imaging sequence (voxel size 3.4 × 3.4 × 4.0 mm^3^, TR = 2.0 s, TE = 30 ms, flip angle 77°, TA = 10 min). During the rs-fMRI the subjects closed their eyes but were not allowed to sleep. All images covered the whole brain including the cerebellum.

### Image processing

Pre-processing steps were performed using SPM (version SPM12; Wellcome Trust Centre for Neuroimaging, University College London, UK; www.fil.ion.ucl.ac.uk/spm/software/spm12/) unless mentioned otherwise. Resting state fMRI Nifti images were realigned and slice time corrected. The mean functional image and the structural MRI were co-registered using normalized mutual information. Next, we segmented the structural MRI and during this process, the forward deformation field to MNI space was determined. We applied this deformation field to the realigned and slice time corrected functional images to warp them to MNI using a voxel size of 2 × 2 × 2 mm^3^ (Sendai) or 3 × 3 × 3 mm^3^ (Goteborg). The segmentations were also warped in the same way but with a voxel size of 1 × 1 × 1 mm^3^.

Based on the motion regressors, we identified censored volumes defined as volumes which had a translation >1 mm or rotation > 1° over the run (per direction) or a scan to scan framewise displacement (FD) > 1 mm. Scan to scan displacement was defined as

$$FD=\mathop{\sum }\limits_{i=1}^{3}|\Delta {x}_{i}|+50\mathop{\sum }\limits_{i=1}^{3}|\Delta {\theta }_{i}|$$

in which displacements $\Delta x$ are expressed^51^ in mm and rotations $\Delta \theta$ in radians. Subjects were only included if there was an interval of at least 5 min in which at most 10% of the data are censored.

The spatially normalized time series (excluding censored volumes) were linearly detrended and two physiological noise regressors (average time series in WM and CSF) were extracted. We also extracted a global signal regressor (averaged signal across all voxels within the brain mask defined as GM + WM + CSF > 0.9) in which GM, WM and CSF are the fuzzy segmentation maps.

The functional data (excluding censored volumes) were then corrected by regressing out these physiological noise regressors as well as the 6 motion regressors obtained during the realignment step.

Then we performed a band-pass filtering (0.009–0.1 Hz) of the functional data using an in-house developed script in which we replaced censored data points by interpolated ones before band-pass filtering but afterwards these volumes were censored again.

### Node definition

We defined 45 regions of interest (see Table 2) which consisted of the regions of the default mode network and regions from the so-called pain matrix. All nodes were present in both the right and left hemisphere, i.e. they had a homologue counterpart in the other hemisphere, except for the periaqueductal gray (PAG) as PAG is in the middle of the brainstem.

The list of regions of the DMN were taken from^52^ and we used the Destrieux atlas^53^ to define these regions in MNI space. The list of regions from the pain matrix were based on a number of published papers^9,54–56^. We used the following atlases to define these regions in MNI space: the Destrieux atlas^53^, the AAL atlas^57^ available in MRIcron (https://www.nitrc.org/projects/mricron) and the Brodmann atlas available in MRIcron. We used an insular subdivision into anterior, middle and posterior insula which was obtained from the UCLA group^58^. For the PAG we used a sphere of 6 mm radius around the MNI coordinate 0,−28,−8^9^. We verified that regions were not overlapping. These regions were taken as the nodes of the network (Fig. 1).

### Functional connectivity

In each node, we extracted the average corrected time series. The averaging was performed across all GM voxels in the node (i.e. voxels in the node in which the GM segmentation was more than 0.3. Note that in this way, the averaging was subject specific and could capture (at least partly) the subject specific functional data in that node. Then we calculated the partial correlations among all pairs of averaged time series for each subject.

### Graph analysis

To create the weights of the graph (i.e. the connection strength), we selected the absolute value of the partial correlations. Note that the weights are values between 0 and 1 and that negative and positive correlations with similar amplitude would get the same weight in that case. For each subject we obtained a weighted graph in this way. From this graph, we calculated global graph measures (characteristic path length, clustering coefficient, global efficiency, betweenness centrality) as well as local graph measures (node strength, average shortest path length, nodal clustering coefficient, local efficiency, nodal betweenness centrality). These graph measures were calculated using the brain connectivity toolbox^59^ (for weighted graphs) except for the (nodal) clustering coefficient and local efficiency which were calculated using the method described in Wang *et al*.^60^. Since graph measures depend on the weight distribution, we normalized these graph measures by dividing them by the graph measure obtained in 1000 random equivalent graph (i.e. a graph with the same number of nodes and weight distribution but in which the weights are randomly assigned).

We also calculated which nodes were considered as hubs using the hubscore^32,61–63^. The hub score is the sum of the dummy values for four criteria (each set at 1 or 0 depending on whether or not the criterion is fulfilled, with a maximum of 4). These criteria are whether the node belongs to the top 20% of nodes

1. showing the highest degree,
2. showing the lowest path length,
3. showing the lowest local cluster coefficient, and
4. showing the highest betweenness centrality.

When a node had a hub score of 2 or more, it was marked as a hub^14^.

The modularity structure was determined using the algorithm of Newman^64,65^ as implemented in the Brain Connectivity Toolbox to determine the community structure of the network^59^.

### Statistics

Data were analysed using Statistical Analysis System (SAS) version 9.4 (SAS Institute Inc, Cary, NC, USA).

Descriptive subject characteristics were compared between IBS patients and HC within each cohort using Kruskal-Wallis non-parametric one-way analysis of variance (ANOVA) due to the non-normal distributions of some of the variables (with HC scoring consistently very low on symptom measures), except for sex distribution which was compared using a Pearson χ² test.

Global graph measures were compared between IBS patients and HC within each cohort using two-tailed independent samples t-tests (assuming unequal variance).

The probability for a node to be a hub, or for a pair of nodes to belong to the same module, was compared between IBS patients and HC using Fisher exact tests.

The relationship between IBS symptom severity and GI-specific anxiety on the one hand and global graph measures on the other was tested in IBS patients only (given very low values and very low variability in HC) using Spearman correlation analysis given the non-normal distribution of some of the variables under study.

Analysis of covariance (ANCOVA) was used to study the relationship between levels of anxiety and depressive symptoms (total HADS score which will be referred to as “psychological distress”) and global graph measures (main effect of psychological distress) and compare this relationship between groups (group-by-psychological distress interaction effect). The interaction between the continuous covariate (psychological distress) and the dichotomous factor (group) tests the difference in slope (i.e. correlation) for the psychological distress – graph measure relationship between both groups. We omitted global efficiency from this analysis given its strong inverse correlation with characteristic path length. For the purpose of this analysis, Box-Cox transformations^66^ were used to normalize distributions of the dependent variables and/or covariates where needed to fulfil the assumption of normally distributed residuals in ANCOVA, and covariates were standardized with mean 0 and standard deviation 1.

## Supplementary information

Supplementary material.

## Data Availability

The datasets generated during and/or analysed during the current study are available from the corresponding author on reasonable request.
