# Supplementary material for: Resting state functional connectivity of the pain matrix and default mode network in irritable bowel syndrome: a graph theoretical analysis
Source: Sci Rep. 2020 Jul 3;10:11015. doi: 10.1038/s41598-020-67048-9 (PMC7335204; doi:10.1038/s41598-020-67048-9)
Supplement: Supplementary file 1 — Supplementary material. [file 41598_2020_67048_MOESM1_ESM.docx]

Resting state functional connectivity of the pain matrix and default mode network in irritable bowel syndrome: a graph theoretical analysis

Michiko Kano^1,2^, Cecilia Grinsvall^3^, Qian Ran^4^, Patrick Dupont^4^, Joe Morishita^2^, Tomohiko Muratsubaki^2^, Shunji Mugikura^5^, Huynh Giao Ly^6^, Hans Törnblom^3^, Maria Ljungberg^7,8^, Kei Takase^5^, Magnus Simrén^3^, Lukas Van Oudenhove^6,9^, Shin Fukudo^2^

^1^Sukawa clinic, Kirari health coop, Fukushima, Japan

^2^Behavioral Medicine, Graduate School of Medicine, Tohoku University, Sendai, Japan

^3^Department of Internal Medicine & Clinical Nutrition, Institute of Medicine, Sahlgrenska Academy, University of Gothenburg, Gothenburg, Sweden

^4^Laboratory for Cognitive Neurology, KU Leuven, Leuven, Belgium

^5^ Diagnostic Radiology, Tohoku University Hospital, Sendai, Japan

^6^ Laboratory for Brain-Gut Axis Studies (LaBGAS), Translational Research Center for Gastrointestinal Disorders (TARGID), KU Leuven, Leuven, Belgium

^7^ Department of Radiation Physics, Institute of Clinical Sciences, Sahlgrenska Academy, University of Gothenburg, Gothenburg, Sweden.

^8^ Department of Medical Physics and Biomedical Engineering, Diagnostic Imaging, Sahlgrenska University Hospital, MR Centre, Gothenburg, Sweden.

^9^ Cognitive and Affective Neuroscience Lab, Department of Psychological & Brain Sciences, Dartmouth College, Hanover, NH, USA

Supplementary material

## ***Significant differences in modularity structure between IBS patients and healthy controls in pain matrix and DMN***

## ***Table S1.***

### *a. Sendai cohort*

| **node 1** | **node 2** | **probability in HC (%)** | **probability in IBS (%)** | **p-value (uncorrected)** |
| --- | --- | --- | --- | --- |
| L amygdala | L angular gyrus | 24.14 | 6.67 | 0.028 |
| L amygdala | L anterior insula | 27.59 | 10 | 0.038 |
| L amygdala | L posterior insula | 31.03 | 13.33 | 0.047 |
| L amygdala | L mPFC | 31.03 | 10 | 0.019 |
| L amygdala | L pACC | 3.45 | 23.33 | 0.009 |
| L amygdala | L parahippocampal gyrus | 10.34 | 36.67 | 0.006 |
| L amygdala | R anterior insula | 37.93 | 13.33 | 0.012 |
| L amygdala | R parahippocampal gyrus | 31.03 | 13.33 | 0.047 |
| L angular gyrus | L anterior insula | 44.83 | 10 | < 0.001 |
| L angular gyrus | L IPL | 24.14 | 53.33 | 0.008 |
| L angular gyrus | L thalamus | 13.79 | 36.67 | 0.018 |
| L angular gyrus | R LTC | 37.93 | 16.67 | 0.030 |
| L angular gyrus | R SI | 6.9 | 23.33 | 0.035 |
| L angular gyrus | R thalamus | 3.45 | 36.67 | < 0.001 |
| L angular gyrus | R parahippocampal gyrus | 27.59 | 10 | 0.038 |
| L hippocampus | L thalamus | 27.59 | 10 | 0.038 |
| L hippocampus | PAG | 27.59 | 10 | 0.038 |
| L hippocampus | R middle insula | 10.34 | 26.67 | 0.049 |
| L hippocampus | R pACC | 31.03 | 10 | 0.019 |
| L hippocampus | R precuneus | 6.9 | 23.33 | 0.035 |
| L anterior insula | L SII | 37.93 | 16.67 | 0.030 |
| L anterior insula | L dlPFC | 31.03 | 13.33 | 0.047 |
| L anterior insula | R angular gyrus | 44.83 | 10 | < 0.001 |
| L anterior insula | R PCC | 27.59 | 6.67 | 0.014 |
| L anterior insula | R parahippocampal gyrus | 31.03 | 13.33 | 0.047 |
| L anterior insula | R sACC | 37.93 | 13.33 | 0.012 |
| L anterior insula | R vlPFC | 13.79 | 36.67 | 0.018 |
| L middle insula | R vlPFC | 31.03 | 13.33 | 0.047 |
| L posterior insula | L PCC | 27.59 | 6.67 | 0.014 |
| L posterior insula | L SII | 17.24 | 40 | 0.023 |
| L posterior insula | L aMCC | 31.03 | 10 | 0.019 |
| L IPL | L dlPFC | 6.9 | 23.33 | 0.035 |
| L IPL | L pACC | 3.45 | 26.67 | 0.004 |
| L IPL | R angular gyrus | 13.79 | 36.67 | 0.018 |
| L IPL | R middle insula | 27.59 | 10 | 0.038 |
| L IPL | R LTC | 3.45 | 23.33 | 0.009 |
| L IPL | R pACC | 3.45 | 26.67 | 0.004 |
| L LTC | R anterior insula | 27.59 | 10 | 0.038 |
| L LTC | R dlPFC | 31.03 | 13.33 | 0.047 |
| L PCC | L SI | 10.34 | 26.67 | 0.049 |
| L PCC | R IPL | 6.9 | 30 | 0.008 |
| L PCC | R thalamus | 31.03 | 13.33 | 0.047 |
| L putamen | L pMCC | 10.34 | 26.67 | 0.049 |
| L putamen | R middle insula | 27.59 | 6.67 | 0.014 |
| L putamen | R dlPFC | 6.9 | 23.33 | 0.035 |
| L putamen | R pMCC | 10.34 | 26.67 | 0.049 |
| L putamen | R sACC | 31.03 | 13.33 | 0.047 |
| L putamen | R vlPFC | 10.34 | 33.33 | 0.013 |
| L SI | L thalamus | 27.59 | 10 | 0.038 |
| L SI | L dlPFC | 31.03 | 10 | 0.019 |
| L SI | PAG | 10.34 | 43.33 | < 0.001 |
| L SI | R anterior insula | 10.34 | 26.67 | 0.049 |
| L SI | R thalamus | 27.59 | 6.67 | 0.014 |
| L SI | R aMCC | 27.59 | 10 | 0.038 |
| L SII | L dlPFC | 41.38 | 10 | 0.002 |
| L SII | R angular gyrus | 31.03 | 10 | 0.019 |
| L SII | R hippocampus | 37.93 | 13.33 | 0.012 |
| L SII | R pMCC | 10.34 | 30 | 0.026 |
| L thalamus | L precuneus | 31.03 | 13.33 | 0.047 |
| L thalamus | R vlPFC | 6.9 | 26.67 | 0.017 |
| L aMCC | L pMCC | 41.38 | 20 | 0.034 |
| L aMCC | R amygdala | 27.59 | 10 | 0.038 |
| L aMCC | R SI | 31.03 | 13.33 | 0.047 |
| L aMCC | R aMCC | 48.28 | 70 | 0.041 |
| L aMCC | R pMCC | 24.14 | 6.67 | 0.028 |
| L dlPFC | L precuneus | 10.34 | 33.33 | 0.013 |
| L dlPFC | R IPL | 10.34 | 26.67 | 0.049 |
| L dlPFC | R PCC | 10.34 | 33.33 | 0.013 |
| L dlPFC | R precuneus | 10.34 | 26.67 | 0.049 |
| L dlPFC | R sACC | 41.38 | 6.67 | < 0.001 |
| L dlPFC | R vlPFC | 17.24 | 3.33 | 0.037 |
| L mPFC | L pACC | 17.24 | 3.33 | 0.036 |
| L mPFC | R hippocampus | 27.59 | 10 | 0.038 |
| L mPFC | R IPL | 37.93 | 16.67 | 0.030 |
| L mPFC | R pACC | 24.14 | 0 | 0.001 |
| L pACC | R amygdala | 10.34 | 26.67 | 0.049 |
| L pACC | R angular gyrus | 13.79 | 36.67 | 0.018 |
| L pACC | R posterior insula | 24.14 | 3.33 | 0.008 |
| L pACC | R LTC | 37.93 | 16.67 | 0.030 |
| L pACC | R pMCC | 27.59 | 10 | 0.038 |
| L pACC | R precuneus | 24.14 | 6.67 | 0.028 |
| L pMCC | PAG | 31.03 | 13.33 | 0.047 |
| L pMCC | R putamen | 10.34 | 33.33 | 0.013 |
| L pMCC | R SI | 24.14 | 6.67 | 0.028 |
| L pMCC | R vlPFC | 41.38 | 6.67 | < 0.001 |
| L parahippocampal gyrus | R amygdala | 13.79 | 46.67 | 0.002 |
| L precuneus | L sACC | 10.34 | 33.33 | 0.013 |
| L precuneus | R pMCC | 31.03 | 6.67 | 0.006 |
| L precuneus | R parahippocampal gyrus | 31.03 | 10 | 0.019 |
| L sACC | R precuneus | 10.34 | 30 | 0.026 |
| L vlPFC | R hippocampus | 41.38 | 10 | 0.002 |
| L vlPFC | R middle insula | 6.9 | 26.67 | 0.017 |
| L vlPFC | R IPL | 37.93 | 10 | 0.004 |
| L vlPFC | R mPFC | 27.59 | 10 | 0.038 |
| PAG | R vlPFC | 27.59 | 10 | 0.038 |
| R amygdala | R posterior insula | 6.9 | 23.33 | 0.035 |
| R amygdala | R LTC | 31.03 | 13.33 | 0.047 |
| R amygdala | R pACC | 10.34 | 26.67 | 0.049 |
| R angular gyrus | R IPL | 10.34 | 46.67 | < 0.001 |
| R angular gyrus | R SI | 13.79 | 33.33 | 0.034 |
| R angular gyrus | R SII | 24.14 | 6.67 | 0.028 |
| R angular gyrus | R thalamus | 10.34 | 26.67 | 0.049 |
| R angular gyrus | R dlPFC | 44.83 | 13.33 | 0.002 |
| R angular gyrus | R pACC | 3.45 | 30 | 0.002 |
| R angular gyrus | R parahippocampal gyrus | 31.03 | 13.33 | 0.047 |
| R hippocampus | R IPL | 27.59 | 6.67 | 0.014 |
| R hippocampus | R SII | 34.48 | 10 | 0.009 |
| R hippocampus | R vlPFC | 37.93 | 16.67 | 0.030 |
| R anterior insula | R middle insula | 13.79 | 43.33 | 0.004 |
| R anterior insula | R mPFC | 27.59 | 10 | 0.038 |
| R anterior insula | R vlPFC | 13.79 | 33.33 | 0.034 |
| R middle insula | R aMCC | 27.59 | 10 | 0.038 |
| R IPL | R thalamus | 20.69 | 46.67 | 0.014 |
| R IPL | R parahippocampal gyrus | 3.45 | 23.33 | 0.009 |
| R IPL | R precuneus | 24.14 | 6.67 | 0.028 |
| R LTC | R SII | 31.03 | 13.33 | 0.047 |
| R LTC | R dlPFC | 20.69 | 0 | 0.003 |
| R LTC | R pACC | 37.93 | 16.67 | 0.030 |
| R LTC | R vlPFC | 24.14 | 46.67 | 0.031 |
| R PCC | R putamen | 31.03 | 13.33 | 0.047 |
| R putamen | R pMCC | 6.9 | 36.67 | 0.001 |
| R putamen | R sACC | 37.93 | 13.33 | 0.012 |
| R putamen | R vlPFC | 3.45 | 30 | 0.002 |
| R SI | R thalamus | 37.93 | 16.67 | 0.030 |
| R SI | R aMCC | 31.03 | 10 | 0.019 |
| R SII | R vlPFC | 31.03 | 13.33 | 0.047 |
| R thalamus | R precuneus | 31.03 | 13.33 | 0.047 |
| R thalamus | R vlPFC | 3.45 | 16.67 | 0.041 |
| R aMCC | R mPFC | 17.24 | 36.67 | 0.042 |
| R aMCC | R vlPFC | 20.69 | 0 | 0.003 |
| R dlPFC | R sACC | 37.93 | 6.67 | < 0.001 |
| R pACC | R precuneus | 34.48 | 10 | 0.009 |
| R pACC | R sACC | 10.34 | 33.33 | 0.013 |

### *b. Gothenburg cohort*

| **node 1** | **node 2** | **probability in HC (%)** | **probability in IBS (%)** | **p-value (uncorrected)** |
| --- | --- | --- | --- | --- |
| L amygdala | L aMCC | 10.34 | 24.19 | 0.039 |
| L amygdala | L parahippocampal gyrus | 27.59 | 11.29 | 0.039 |
| L amygdala | R posterior insula | 6.90 | 20.97 | 0.022 |
| L amygdala | R LTC | 13.79 | 30.65 | 0.026 |
| L amygdala | R dlPFC | 37.93 | 17.74 | 0.024 |
| L amygdala | R pACC | 27.59 | 11.29 | 0.039 |
| L angular gyrus | L SII | 13.79 | 29.03 | 0.038 |
| L angular gyrus | R amygdala | 34.48 | 17.74 | 0.048 |
| L angular gyrus | R dlPFC | 48.28 | 24.19 | 0.013 |
| L angular gyrus | R mPFC | 37.93 | 12.90 | 0.006 |
| L angular gyrus | R precuneus | 27.59 | 11.29 | 0.039 |
| L hippocampus | L LTC | 6.90 | 24.19 | 0.008 |
| L hippocampus | L precuneus | 31.03 | 8.06 | 0.007 |
| L hippocampus | R hippocampus | 58.62 | 37.10 | 0.025 |
| L hippocampus | R precuneus | 27.59 | 11.29 | 0.039 |
| L anterior insula | L posterior insula | 3.45 | 17.74 | 0.008 |
| L anterior insula | L LTC | 10.34 | 33.87 | 0.002 |
| L anterior insula | L vlPFC | 10.34 | 24.19 | 0.039 |
| L anterior insula | R putamen | 13.79 | 30.65 | 0.026 |
| L anterior insula | R aMCC | 6.90 | 22.58 | 0.014 |
| L anterior insula | R mPFC | 41.38 | 22.58 | 0.038 |
| L anterior insula | R sACC | 34.48 | 14.52 | 0.022 |
| L middle insula | R amygdala | 44.83 | 17.74 | 0.005 |
| L middle insula | R IPL | 10.34 | 24.19 | 0.039 |
| L posterior insula | L IPL | 6.90 | 24.19 | 0.008 |
| L posterior insula | L putamen | 24.14 | 9.68 | 0.050 |
| L posterior insula | L thalamus | 31.03 | 12.90 | 0.029 |
| L posterior insula | L aMCC | 6.90 | 20.97 | 0.022 |
| L posterior insula | R posterior insula | 27.59 | 46.77 | 0.033 |
| L posterior insula | R putamen | 34.48 | 11.29 | 0.008 |
| L posterior insula | R aMCC | 6.90 | 20.97 | 0.022 |
| L posterior insula | R mPFC | 10.34 | 27.42 | 0.016 |
| L IPL | L pMCC | 31.03 | 14.52 | 0.044 |
| L IPL | R IPL | 41.38 | 59.68 | 0.049 |
| L IPL | R mPFC | 41.38 | 14.52 | 0.004 |
| L IPL | R pACC | 27.59 | 11.29 | 0.039 |
| L LTC | L SI | 13.79 | 33.87 | 0.011 |
| L LTC | L pMCC | 10.34 | 27.42 | 0.016 |
| L LTC | L parahippocampal gyrus | 10.34 | 27.42 | 0.016 |
| L LTC | R hippocampus | 10.34 | 24.19 | 0.039 |
| L LTC | R middle insula | 24.14 | 3.23 | 0.006 |
| L LTC | R dlPFC | 41.38 | 16.13 | 0.007 |
| L LTC | R precuneus | 37.93 | 19.35 | 0.034 |
| L PCC | L putamen | 13.79 | 32.26 | 0.017 |
| L PCC | L SI | 6.90 | 19.35 | 0.035 |
| L PCC | L mPFC | 17.24 | 33.87 | 0.036 |
| L PCC | R angular gyrus | 34.48 | 17.74 | 0.048 |
| L PCC | R posterior insula | 34.48 | 16.13 | 0.033 |
| L putamen | L dlPFC | 3.45 | 20.97 | 0.002 |
| L putamen | R anterior insula | 10.34 | 25.81 | 0.026 |
| L putamen | R dlPFC | 3.45 | 20.97 | 0.002 |
| L putamen | R pMCC | 31.03 | 14.52 | 0.044 |
| L putamen | R parahippocampal gyrus | 31.03 | 9.68 | 0.011 |
| L putamen | R precuneus | 3.45 | 16.13 | 0.014 |
| L SI | L pACC | 31.03 | 14.52 | 0.044 |
| L SI | L pMCC | 10.34 | 25.81 | 0.026 |
| L SI | R middle insula | 37.93 | 16.13 | 0.016 |
| L SI | R posterior insula | 10.34 | 24.19 | 0.039 |
| L SI | R SI | 79.31 | 58.06 | 0.015 |
| L SI | R parahippocampal gyrus | 10.34 | 25.81 | 0.026 |
| L SII | L pMCC | 34.48 | 16.13 | 0.033 |
| L SII | R amygdala | 34.48 | 14.52 | 0.022 |
| L SII | R dlPFC | 3.45 | 17.74 | 0.008 |
| L thalamus | R putamen | 31.03 | 14.52 | 0.044 |
| L thalamus | R parahippocampal gyrus | 3.45 | 20.97 | 0.002 |
| L aMCC | R PCC | 37.93 | 16.13 | 0.016 |
| L dlPFC | R posterior insula | 24.14 | 9.68 | 0.050 |
| L dlPFC | R putamen | 3.45 | 22.58 | 0.001 |
| L mPFC | L parahippocampal gyrus | 37.93 | 14.52 | 0.010 |
| L mPFC | L precuneus | 37.93 | 19.35 | 0.036 |
| L mPFC | R IPL | 31.03 | 14.52 | 0.044 |
| L mPFC | R pMCC | 17.24 | 37.10 | 0.017 |
| L mPFC | R precuneus | 34.48 | 12.90 | 0.014 |
| L pACC | L pMCC | 34.48 | 16.13 | 0.033 |
| L pACC | L precuneus | 6.90 | 20.97 | 0.022 |
| L pACC | R middle insula | 6.90 | 19.35 | 0.035 |
| L pACC | R IPL | 41.38 | 12.90 | 0.002 |
| L pACC | R PCC | 13.79 | 29.03 | 0.038 |
| L pACC | R SII | 6.90 | 20.97 | 0.022 |
| L pACC | R precuneus | 6.90 | 19.35 | 0.035 |
| L pACC | R sACC | 6.90 | 20.97 | 0.022 |
| L pMCC | R SI | 10.34 | 24.19 | 0.039 |
| L pMCC | R parahippocampal gyrus | 10.34 | 33.87 | 0.002 |
| L pMCC | R sACC | 34.48 | 16.13 | 0.033 |
| L parahippocampal gyrus | R posterior insula | 6.90 | 19.35 | 0.035 |
| L parahippocampal gyrus | R LTC | 3.45 | 20.97 | 0.002 |
| L parahippocampal gyrus | R PCC | 31.03 | 14.52 | 0.044 |
| L parahippocampal gyrus | R sACC | 13.79 | 30.65 | 0.026 |
| L precuneus | R hippocampus | 34.48 | 17.74 | 0.048 |
| L sACC | R middle insula | 13.79 | 32.26 | 0.017 |
| L sACC | R IPL | 10.34 | 29.03 | 0.010 |
| L sACC | R LTC | 34.48 | 14.52 | 0.022 |
| L sACC | R thalamus | 34.48 | 14.52 | 0.022 |
| L sACC | R aMCC | 6.90 | 24.19 | 0.008 |
| L sACC | R parahippocampal gyrus | 34.48 | 11.29 | 0.008 |
| L sACC | R vlPFC | 6.90 | 20.97 | 0.022 |
| L vlPFC | R LTC | 13.79 | 33.87 | 0.011 |
| L vlPFC | R mPFC | 10.34 | 24.19 | 0.039 |
| PAG | R LTC | 6.90 | 35.48 | < 0.001 |
| PAG | R putamen | 34.48 | 16.13 | 0.033 |
| R amygdala | R aMCC | 37.93 | 19.35 | 0.036 |
| R amygdala | R pACC | 37.93 | 12.90 | 0.006 |
| R amygdala | R sACC | 34.48 | 14.52 | 0.022 |
| R angular gyrus | R SI | 10.34 | 25.81 | 0.026 |
| R angular gyrus | R mPFC | 31.03 | 11.29 | 0.019 |
| R hippocampus | R IPL | 13.79 | 29.03 | 0.038 |
| R hippocampus | R mPFC | 13.79 | 29.03 | 0.038 |
| R anterior insula | R posterior insula | 37.93 | 16.13 | 0.016 |
| R middle insula | R pACC | 3.45 | 16.13 | 0.014 |
| R middle insula | R sACC | 13.79 | 29.03 | 0.038 |
| R posterior insula | R LTC | 34.48 | 12.90 | 0.014 |
| R posterior insula | R PCC | 34.48 | 16.13 | 0.033 |
| R posterior insula | R SI | 6.90 | 20.97 | 0.022 |
| R posterior insula | R dlPFC | 27.59 | 9.68 | 0.025 |
| R IPL | R putamen | 6.90 | 20.97 | 0.022 |
| R IPL | R mPFC | 34.48 | 17.74 | 0.048 |
| R LTC | R SI | 13.79 | 30.65 | 0.026 |
| R LTC | R dlPFC | 34.48 | 17.74 | 0.048 |
| R PCC | R putamen | 31.03 | 8.06 | 0.007 |
| R SI | R parahippocampal gyrus | 10.34 | 25.81 | 0.026 |
| R thalamus | R pACC | 34.48 | 11.29 | 0.008 |
| R thalamus | R parahippocampal gyrus | 6.90 | 20.97 | 0.022 |
| R mPFC | R precuneus | 27.59 | 8.06 | 0.015 |
| R mPFC | R vlPFC | 3.45 | 20.97 | 0.002 |
| R pMCC | R sACC | 10.34 | 27.42 | 0.016 |
| R precuneus | R vlPFC | 6.90 | 24.19 | 0.008 |

HC, healthy controls; IBS, irritable bowel syndrome; L, left; R, right; IPL, inferior parietal lobule; LTC, lateral temporal cortex; PCC, posterior cingulate cortex; SI/SII, primary/secondary somatosensory cortex; aMCC, anterior midcingulate cortex; dlPFC, dorsolateral prefrontal cortex; mPFC, medial prefrontal cortex; pACC, pregenual anterior cingulate cortex; pMCC, posterior midcingulate cortex; sACC, subgenual anterior cingulate cortex; vlPFC, ventrolateral prefrontal cortex; PAG, periaqueductal grey matter

## ***2. Whole brain analysis using the Shen 50 atlas***

## ***Table S2.*** *Normalized global graph measures in IBS patients compared to healthy controls*

### *Sendai cohort*

| **Normalized graph measure** | **healthy controls** | **IBS patients** | **p-value** |
| --- | --- | --- | --- |
| clustering coefficient | 1.0092 ± 0.0004 | 1.0092 ± 0.0003 | 0.49 |
| efficiency | 0.9698 ± 0.0008 | 0.9686 ± 0.0011 | 0.25 |
| betweenness centrality | 0.9828 ± 0.0021 | 0.981 ± 0.0025 | 0.35 |
| characteristic path length | 1.0402 ± 0.0011 | 1.0418 ± 0.0016 | 0.30 |

### *Gothenburg cohort*

| **Normalized graph measure** | **healthy controls** | **IBS patients** | **p-value** |
| --- | --- | --- | --- |
| clustering coefficient | 1.0081 ± 0.00264 | 1.0079 ± 0.000235 | 0.77 |
| efficiency | 0.9758 ± 0.000925 | 0.9763 ± 0.000552 | 0.60 |
| betweenness centrality | 0.9871 ± 0.00164 | 0.9889 ± 0.00133 | 0.42 |
| characteristic path length | 1.0331 ± 0.00142 | 1.0320 ± 0.000759 | 0.50 |

Values are averages (±SEM) of normalized graph measures; p-values from independent samples t-tests

IBS, irritable bowel syndrome

## ***Table S3.*** *Overview of hubs in IBS patients and healthy controls*

### *Sendai cohort*

| **healthy controls** | **IBS patients** |
| --- | --- |
| Precentral_R/Frontal_Inf_Oper_R | Occipital_Inf_R/Occipital_Mid_R |
| SupraMarginal_R/Parietal_Inf_R | Cuneus_R/Precuneus_R |
| Occipital_Inf_R/Occipital_Mid_R | Lingual_R/Calcarine_R |
| Parietal_Sup_R/Angular_R | Occipital_Inf_R/Temporal_Inf_R |
| SupraMarginal_L/Parietal_Inf_L | Temporal_Mid_R/Angular_R |
| Cuneus_L/Occipital_Sup_L | Postcentral_L/Parietal_Inf_L |
|  | Angular_L/Temporal_Mid_L |

### *Gothenburg cohort*

| **healthy controls** | **IBS patients** |
| --- | --- |
| Frontal_Inf_Orb_R | Frontal_Sup_Orb_R/Frontal_Mid_Orb_R |
| Parietal_Sup_L/Parietal_Inf_L | Frontal_Mid_R/Frontal_Sup_R |
|  | Cerebelum_Crus2_R/Cerebelum_Crus1_R |
|  | Frontal_Inf_Orb_L/Frontal_Inf_Tri_L |
|  | Cerebelum_Crus1_L/Cerebelum_Crus2_L |
|  | Parietal_Sup_L/Parietal_Inf_L |
|  | Frontal_Sup_Medial_L/Frontal_Sup_L |
|  | Frontal_Inf_Tri_L |

IBS, irritable bowel syndrome; L, left; R, right; Inf, inferior; Mid, middle; Sup, superior; Oper, opercular; Orb, orbital; Tri, triangular; Labels are assigned using the AAL atlas.

## ***Table S4.*** *Significant differences in probability to be a hub between IBS patients and healthy controls*

### *Sendai cohort*

| **node** | **% hub in healthy controls** | **% hub in IBS patients** | **p-value (uncorrected)** |
| --- | --- | --- | --- |
| Precentral_R/Frontal_Inf_Oper_R | 45 | 17 | 0.009 |
| SupraMarginal_R/Parietal_Inf_R | 48 | 14 | 0.001 |
| Cerebelum_8_R/Cerebelum_9_R | 7 | 24 | 0.031 |
| Caudate_R | 28 | 10 | 0.034 |
| Thalamus_R | 21 | 40 | 0.007 |
| Occipital_Mid_L/Calcarine_L | 7 | 57 | <0.0001* |
| Angular_L/Temporal_Mid_L | 28 | 62 | 0.002 |
| ParaHippocampal_L/Hippocampus_L ParaHippocampal_L/Hippocampus_L  ParaHippocampal_L/Hippocampus_L  ParaHippocampal_L/Hippocampus_L | 28 | 3 | 0.004 |

### *Gothenburg cohort*

| **node** | **% hub in healthy controls** | **% hub in IBS patients** | **p-value (uncorrected)** |
| --- | --- | --- | --- |
| Cerebelum_4_5_R/Cerebelum_6_R  Cerebelum_4_5_R/Cerebelum_6_R | 20.69 | 3.23 | 0.013 |
| Precentral_R/Frontal_Sup_R | 10.34 | 29.03 | 0.010 |
| Precuneus_R/Cingulum_Post_R  Precuneus_R/Cingulum_Post_R | 41.38 | 17.74 | 0.011 |
| ParaHippocampal_R/Hippocampus_R | 20.69 | 6.45 | 0.04 |
| Precuneus_R | 34.48 | 14.52 | 0.022 |
| Cerebelum_6_R/Cerebelum_Crus1_R  Cerebelum_6_R/Cerebelum_Crus1_R  Cerebelum_6_R/Cerebelum_Crus1_R  Cerebelum_6_R/Cerebelum_Crus1_R  Cerebelum_6_R/Cerebelum_Crus1_R | 6.90 | 24.19 | 0.008 |
| Thalamus_R | 13.79 | 1.61 | 0.033 |
| Frontal_Mid_L | 44.83 | 25.81 | 0.038 |
| Putamen_L | 3.45 | 14.52 | 0.024 |
| Cerebelum_8_L | 10.34 | 30.65 | 0.006 |
| Occipital_Inf_L/Occipital_Mid_L  Occipital_Inf_L/Occipital_Mid_L  Occipital_Inf_L/Occipital_Mid_L | 3.45 | 25.81 | 0.0003* |

* Significant after FDR correction for multiple testing

IBS, irritable bowel syndrome; L, left; R, right; Inf, inferior; Mid, middle; Sup, superior; Oper, opercular; Labels are assigned using the AAL atlas.

## ***Table S5.*** *Modularity structure in IBS patients and healthy controls*

### *Sendai cohort*

**HC**

| Cerebelum_Crus2_R/Cerebelum_8_R | Frontal_Sup_Orb_R/Frontal_Mid_Orb_R | Precentral_R/Frontal_Inf_Oper_R |
| --- | --- | --- |
| Cerebelum_4_5_R/Cerebelum_6_R | Frontal_Mid_R/Frontal_Sup_R | SupraMarginal_R/Parietal_Inf_R |
| Occipital_Inf_R/Occipital_Mid_R | Temporal_Sup_R/Rolandic_Oper_R | Temporal_Pole_Mid_R/Temporal_Inf_R |
| Cingulum_Mid_R/Supp_Motor_Area_R | Postcentral_R/SupraMarginal_R | Cingulum_Mid_R/Cingulum_Ant_R |
| Cerebelum_8_R/Cerebelum_9_R | Frontal_Sup_Medial_R | Frontal_Sup_R/Frontal_Mid_R |
| Cuneus_R/Precuneus_R | Precentral_R/Frontal_Sup_R | Temporal_Mid_R |
| Rectus_R/Frontal_Sup_Orb_R | Occipital_Mid_R | Caudate_R |
| Lingual_R/Calcarine_R | Fusiform_R/Hippocampus_R | Temporal_Mid_R/Temporal_Inf_R |
| Cingulum_Ant_R/Frontal_Mid_Orb_R | Postcentral_R/Paracentral_Lobule_R | Temporal_Mid_R/Angular_R |
| Parietal_Sup_R/Angular_R | Frontal_Inf_Oper_R/Frontal_Inf_Tri_R | Insula_R/Frontal_Inf_Orb_R |
| Precuneus_R/Cingulum_Post_R | Postcentral_R | Frontal_Mid_R |
| ParaHippocampal_R/Hippocampus_R | Frontal_Inf_Orb_R | Thalamus_R |
| Occipital_Inf_R/Temporal_Inf_R | Occipital_Mid_L | Frontal_Mid_L |
| Temporal_Sup_R/Insula_R | Frontal_Mid_L/Frontal_Mid_Orb_L | Cerebelum_8_L |
| Precuneus_R | Frontal_Inf_Orb_L/Frontal_Inf_Tri_L | SupraMarginal_L/Parietal_Inf_L |
| Calcarine_R/Cuneus_R | Temporal_Sup_L/Rolandic_Oper_L | Precuneus_L |
| Lingual_R/Cerebelum_6_R | Supp_Motor_Area_L | Cingulum_Mid_L/Cingulum_Ant_L |
| Temporal_Inf_R | Insula_L | ParaHippocampal_L/Hippocampus_L |
| Cerebelum_6_R/Cerebelum_Crus1_R | Temporal_Mid_L/Temporal_Sup_L | Temporal_Mid_L |
| Cerebelum_Crus2_R/Cerebelum_Crus1_R | Postcentral_L/Parietal_Inf_L | Thalamus_L |
| Brainstem R (no AAL) | Postcentral_L | Precuneus_L/Paracentral_Lobule_L |
| Putamen_R/Pallidum_R | Angular_L/Temporal_Mid_L | Caudate_L |
| Rectus_L/Frontal_Mid_Orb_L | Precentral_L/Frontal_Sup_L |  |
| Calcarine_L | Frontal_Sup_Medial_L/Frontal_Sup_L |  |
| Putamen_L | Frontal_Sup_Orb_R/Frontal_Mid_Orb_R |  |
| Cerebelum_Crus2_L/Cerebelum_Crus1_L | Frontal_Mid_R/Frontal_Sup_R |  |
| Occipital_Inf_L/Occipital_Mid_L |  |  |
| Cerebelum_Crus1_L/Cerebelum_Crus2_L |  |  |
| Midbrain L (no AAL) |  |  |
| Cuneus_L/Occipital_Sup_L |  |  |
| Occipital_Mid_L/Calcarine_L |  |  |
| Parietal_Sup_L/Parietal_Inf_L |  |  |
| Cingulum_Ant_L/Frontal_Sup_Medial_L |  |  |
| Cerebelum_6_L/Fusiform_L |  |  |
| Temporal_Inf_L/Temporal_Pole_Mid_L |  |  |
| Temporal_Inf_L/Occipital_Inf_L |  |  |
| Frontal_Inf_Orb_L/Frontal_Sup_Orb_L |  |  |
| Lingual_L/Fusiform_L |  |  |
| Precentral_L/Frontal_Inf_Oper_L |  |  |
| Lingual_L/Cerebelum_6_L |  |  |
| Cerebelum_4_5_L/Cerebelum_6_L |  |  |
| Temporal_Inf_L |  |  |
| Cingulum_Mid_L |  |  |
| Frontal_Inf_Tri_L |  |  |
| Cerebelum_9_L |  |  |
| Brainstem L (no AAL) |  |  |
| Precuneus_L/calcarine_L |  |  |

**IBS**

| Cerebelum_Crus2_R/Cerebelum_8_R | Cerebelum_4_5_R/Cerebelum_6_R | Precentral_R/Frontal_Inf_Oper_R | Temporal_Sup_R/Rolandic_Oper_R |
| --- | --- | --- | --- |
| Frontal_Sup_Orb_R/Frontal_Mid_Orb_R | SupraMarginal_R/Parietal_Inf_R | Frontal_Mid_R/Frontal_Sup_R | Cingulum_Mid_R/Supp_Motor_Area_R |
| Temporal_Pole_Mid_R/Temporal_Inf_R | Occipital_Inf_R/Occipital_Mid_R | Temporal_Mid_R | Cingulum_Mid_R/Cingulum_Ant_R |
| Cerebelum_8_R/Cerebelum_9_R | Postcentral_R/SupraMarginal_R | Frontal_Sup_Medial_R | Cuneus_R/Precuneus_R |
| Rectus_R/Frontal_Sup_Orb_R | Frontal_Sup_R/Frontal_Mid_R | Parietal_Sup_R/Angular_R | Lingual_R/Calcarine_R |
| Caudate_R | Cingulum_Ant_R/Frontal_Mid_Orb_R | Temporal_Mid_R/Temporal_Inf_R | Precuneus_R/Cingulum_Post_R |
| ParaHippocampal_R/Hippocampus_R | Precentral_R/Frontal_Sup_R | Occipital_Mid_R | Calcarine_R/Cuneus_R |
| Temporal_Sup_R/Insula_R | Occipital_Inf_R/Temporal_Inf_R | Fusiform_R/Hippocampus_R | Lingual_R/Cerebelum_6_R |
| Brainstem R (no AAL) | Temporal_Mid_R/Angular_R | Temporal_Inf_R | Insula_R/Frontal_Inf_Orb_R |
| Putamen_R/Pallidum_R | Precuneus_R | Frontal_Mid_R | Cerebelum_6_R/Cerebelum_Crus1_R |
| Putamen_L | Postcentral_R | Thalamus_R | Postcentral_R/Paracentral_Lobule_R |
| Cerebelum_8_L | Occipital_Mid_L | Frontal_Inf_Orb_R | Frontal_Inf_Oper_R/Frontal_Inf_Tri_R |
| Frontal_Inf_Orb_L/Frontal_Inf_Tri_L | Frontal_Mid_L | Frontal_Mid_L/Frontal_Mid_Orb_L | Cerebelum_Crus2_R/Cerebelum_Crus1_R |
| Cerebelum_Crus1_L/Cerebelum_Crus2_L | Temporal_Sup_L/Rolandic_Oper_L | Parietal_Sup_L/Parietal_Inf_L | Rectus_L/Frontal_Mid_Orb_L |
| Insula_L | SupraMarginal_L/Parietal_Inf_L | Cerebelum_6_L/Fusiform_L | Calcarine_L |
| Midbrain L (no AAL) | Occipital_Inf_L/Occipital_Mid_L | Temporal_Inf_L/Occipital_Inf_L | Cerebelum_Crus2_L/Cerebelum_Crus1_L |
| Temporal_Mid_L/Temporal_Sup_L | Cingulum_Ant_L/Frontal_Sup_Medial_L | Frontal_Sup_Medial_L/Frontal_Sup_L | Supp_Motor_Area_L |
| ParaHippocampal_L/Hippocampus_L | Postcentral_L | Temporal_Mid_L | Precuneus_L |
| Temporal_Inf_L/Temporal_Pole_Mid_L | Angular_L/Temporal_Mid_L | Lingual_L/Fusiform_L | Cingulum_Mid_L/Cingulum_Ant_L |
| Frontal_Inf_Orb_L/Frontal_Sup_Orb_L | Precentral_L/Frontal_Sup_L | Temporal_Inf_L | Cuneus_L/Occipital_Sup_L |
| Cerebelum_9_L | Precentral_L/Frontal_Inf_Oper_L | Thalamus_L | Postcentral_L/Parietal_Inf_L |
| Caudate_L | Cerebelum_4_5_L/Cerebelum_6_L |  | Occipital_Mid_L/Calcarine_L |
|  | Frontal_Inf_Tri_L |  | Lingual_L/Cerebelum_6_L |
|  |  |  | Cingulum_Mid_L |
|  |  |  | Brainstem L (no AAL) |
|  |  |  | Precuneus_L/calcarine_L |
|  |  |  | Precuneus_L/Paracentral_Lobule_L |

### *Gothenburg cohort*

**HC**

| Cerebelum_Crus2_R/Cerebelum_8_R | Frontal_Sup_Orb_R/Frontal_Mid_Orb_R | Precentral_R/Frontal_Inf_Oper_R | Occipital_Inf_R/Occipital_Mid_R |
| --- | --- | --- | --- |
| Cerebelum_4_5_R/Cerebelum_6_R | Frontal_Mid_R/Frontal_Sup_R | Temporal_Sup_R/Rolandic_Oper_R | Postcentral_R/SupraMarginal_R |
| Temporal_Pole_Mid_R/Temporal_Inf_R | SupraMarginal_R/Parietal_Inf_R | Rectus_R/Frontal_Sup_Orb_R | Cuneus_R/Precuneus_R |
| Cingulum_Mid_R/Supp_Motor_Area_R | Cingulum_Mid_R/Cingulum_Ant_R | Cingulum_Ant_R/Frontal_Mid_Orb_R | Lingual_R/Calcarine_R |
| Cerebelum_8_R/Cerebelum_9_R | Frontal_Sup_R/Frontal_Mid_R | Temporal_Mid_R/Temporal_Inf_R | Frontal_Sup_Medial_R |
| Temporal_Mid_R | Parietal_Sup_R/Angular_R | Temporal_Sup_R/Insula_R | Precentral_R/Frontal_Sup_R |
| Caudate_R | Precuneus_R/Cingulum_Post_R | Thalamus_R | Occipital_Inf_R/Temporal_Inf_R |
| ParaHippocampal_R/Hippocampus_R | Temporal_Mid_R/Angular_R | Rectus_L/Frontal_Mid_Orb_L | Occipital_Mid_R |
| Fusiform_R/Hippocampus_R | Precuneus_R | SupraMarginal_L/Parietal_Inf_L | Calcarine_R/Cuneus_R |
| Cerebelum_6_R/Cerebelum_Crus1_R | Insula_R/Frontal_Inf_Orb_R | Cingulum_Ant_L/Frontal_Sup_Medial_L | Lingual_R/Cerebelum_6_R |
| Postcentral_R/Paracentral_Lobule_R | Frontal_Mid_R | Precentral_L/Frontal_Inf_Oper_L | Temporal_Inf_R |
| Postcentral_R | Frontal_Inf_Oper_R/Frontal_Inf_Tri_R |  | Calcarine_L |
| Brainstem R (no AAL) | Cerebelum_Crus2_R/Cerebelum_Crus1_R |  | Supp_Motor_Area_L |
| Putamen_R/Pallidum_R | Frontal_Inf_Orb_R |  | Occipital_Inf_L/Occipital_Mid_L |
| Putamen_L | Occipital_Mid_L |  | Cuneus_L/Occipital_Sup_L |
| Cerebelum_8_L | Frontal_Mid_L/Frontal_Mid_Orb_L |  | Occipital_Mid_L/Calcarine_L |
| Temporal_Sup_L/Rolandic_Oper_L | Frontal_Mid_L |  | Postcentral_L |
| Cerebelum_Crus1_L/Cerebelum_Crus2_L | Cerebelum_Crus2_L/Cerebelum_Crus1_L |  | Temporal_Inf_L/Occipital_Inf_L |
| Insula_L | Frontal_Inf_Orb_L/Frontal_Inf_Tri_L |  | Frontal_Sup_Medial_L/Frontal_Sup_L |
| Midbrain L (no AAL) | Precuneus_L |  | Lingual_L/Cerebelum_6_L |
| Temporal_Mid_L/Temporal_Sup_L | Cingulum_Mid_L/Cingulum_Ant_L |  | Temporal_Inf_L |
| Postcentral_L/Parietal_Inf_L | Parietal_Sup_L/Parietal_Inf_L |  | Precuneus_L/calcarine_L |
| Cerebelum_6_L/Fusiform_L | Angular_L/Temporal_Mid_L |  |  |
| ParaHippocampal_L/Hippocampus_L | Temporal_Mid_L |  |  |
| Precentral_L/Frontal_Sup_L | Frontal_Inf_Tri_L |  |  |
| Temporal_Inf_L/Temporal_Pole_Mid_L |  |  |  |
| Frontal_Inf_Orb_L/Frontal_Sup_Orb_L |  |  |  |
| Lingual_L/Fusiform_L |  |  |  |
| Cerebelum_4_5_L/Cerebelum_6_L |  |  |  |
| Cingulum_Mid_L |  |  |  |
| Cerebelum_9_L |  |  |  |
| Brainstem L (no AAL) |  |  |  |
| Thalamus_L |  |  |  |
| Precuneus_L/Paracentral_Lobule_L |  |  |  |
| Caudate_L |  |  |  |

**IBS**

| Cerebelum_Crus2_R/Cerebelum_8_R | Frontal_Sup_Orb_R/Frontal_Mid_Orb_R | SupraMarginal_R/Parietal_Inf_R | Temporal_Sup_R/Rolandic_Oper_R |
| --- | --- | --- | --- |
| Temporal_Pole_Mid_R/Temporal_Inf_R | Cerebelum_4_5_R/Cerebelum_6_R | Occipital_Inf_R/Occipital_Mid_R | Postcentral_R/SupraMarginal_R |
| Cerebelum_8_R/Cerebelum_9_R | Precentral_R/Frontal_Inf_Oper_R | Cingulum_Mid_R/Cingulum_Ant_R | Cingulum_Mid_R/Supp_Motor_Area_R |
| Temporal_Mid_R/Temporal_Inf_R | Frontal_Mid_R/Frontal_Sup_R | Precuneus_R/Cingulum_Post_R | Frontal_Sup_R/Frontal_Mid_R |
| ParaHippocampal_R/Hippocampus_R | Cuneus_R/Precuneus_R | Occipital_Inf_R/Temporal_Inf_R | Rectus_R/Frontal_Sup_Orb_R |
| Temporal_Sup_R/Insula_R | Temporal_Mid_R | Temporal_Mid_R/Angular_R | Cingulum_Ant_R/Frontal_Mid_Orb_R |
| Frontal_Inf_Oper_R/Frontal_Inf_Tri_R | Lingual_R/Calcarine_R | Occipital_Mid_R | Caudate_R |
| Cerebelum_Crus2_R/Cerebelum_Crus1_R | Frontal_Sup_Medial_R | Precuneus_R | Fusiform_R/Hippocampus_R |
| Brainstem R (no AAL) | Parietal_Sup_R/Angular_R | Temporal_Inf_R | Insula_R/Frontal_Inf_Orb_R |
| Cerebelum_8_L | Precentral_R/Frontal_Sup_R | Cerebelum_6_R/Cerebelum_Crus1_R | Postcentral_R/Paracentral_Lobule_R |
| Cerebelum_Crus2_L/Cerebelum_Crus1_L | Calcarine_R/Cuneus_R | Thalamus_R | Putamen_R/Pallidum_R |
| Cerebelum_Crus1_L/Cerebelum_Crus2_L | Lingual_R/Cerebelum_6_R | Frontal_Inf_Orb_R | Rectus_L/Frontal_Mid_Orb_L |
| Temporal_Mid_L/Temporal_Sup_L | Frontal_Mid_R | Occipital_Mid_L | Putamen_L |
| ParaHippocampal_L/Hippocampus_L | Postcentral_R | SupraMarginal_L/Parietal_Inf_L | Temporal_Sup_L/Rolandic_Oper_L |
| Temporal_Inf_L/Temporal_Pole_Mid_L | Frontal_Mid_L/Frontal_Mid_Orb_L | Occipital_Inf_L/Occipital_Mid_L | Supp_Motor_Area_L |
| Cerebelum_9_L | Frontal_Mid_L | Precuneus_L | Insula_L |
| Brainstem L (no AAL) | Calcarine_L | Cingulum_Mid_L/Cingulum_Ant_L | Midbrain L (no AAL) |
|  | Frontal_Inf_Orb_L/Frontal_Inf_Tri_L | Cerebelum_6_L/Fusiform_L | Occipital_Mid_L/Calcarine_L |
|  | Cuneus_L/Occipital_Sup_L | Precentral_L/Frontal_Sup_L | Postcentral_L |
|  | Postcentral_L/Parietal_Inf_L | Temporal_Inf_L/Occipital_Inf_L | Frontal_Inf_Orb_L/Frontal_Sup_Orb_L |
|  | Parietal_Sup_L/Parietal_Inf_L | Temporal_Inf_L | Precentral_L/Frontal_Inf_Oper_L |
|  | Cingulum_Ant_L/Frontal_Sup_Medial_L | Precuneus_L/calcarine_L | Cingulum_Mid_L |
|  | Angular_L/Temporal_Mid_L | Thalamus_L | Precuneus_L/Paracentral_Lobule_L |
|  | Frontal_Sup_Medial_L/Frontal_Sup_L |  | Caudate_L |
|  | Temporal_Mid_L |  |  |
|  | Lingual_L/Fusiform_L |  |  |
|  | Lingual_L/Cerebelum_6_L |  |  |
|  | Cerebelum_4_5_L/Cerebelum_6_L |  |  |
|  | Frontal_Inf_Tri_L |  |  |

IBS, irritable bowel syndrome; L, left; R, right; L, left; R, right; Inf, inferior; Mid, middle; Sup, superior; Oper, opercular; Orb, orbital; Tri, triangular; Supp, supplementary; Labels are assigned using the AAL atlas.

## ***Table S6.*** *Correlations between GI/somatic symptom severity, GI-specific anxiety, and normalized global graph measures*

### *Sendai cohort*

| Normalized graph measure | **IBS-SSS score colonic** | **VSI score** |
| --- | --- | --- |
| clustering coefficient | -0.16 | 0.14 |
| betweenness centrality | 0.29 | 0.24 |
| characteristic path length | -0.23 | -0.19 |

### *Gothenburg cohort*

| Normalized graph measure | **IBS-SSS score colonic** | **IBS-SSS score extra-colonic** | **VSI score** |
| --- | --- | --- | --- |
| clustering coefficient | 0.05 | 0.15 | -0.03 |
| betweenness centrality | 0.21° | 0.26* | -0.006 |
| characteristic path length | -0.13 | -0.05 | 0.02 |

Values are Spearman’s ρ; °p<0.10 (uncorrected); *p<0.05 (uncorrected); ^p<0.05 (FDR corrected)

IBS, irritable bowel syndrome; IBS-SSS, IBS severity scoring system; VSI, visceral sensitivity index

## ***Table S7.*** *Results of ANCOVA analyses testing the relationship between levels of psychological distress and normalized graph measures*

### *Sendai cohort*

|  | **main effect group** | **main effect psychological distress** | **group-by-psychological distress interaction** |
| --- | --- | --- | --- |
| ***trait anxiety*** | | | |
| Normalized clustering coefficient | 0.09 | 0.1 | 1.15 |
| Normalized betweenness centrality | 0.47 | 0.05 | 0.12 |
| Normalized characteristic path length | 1.01* | 0.21 | 0.62 |
| ***depressive symptoms*** | | | |
| Normalized clustering coefficient | 0.02 | 0.35 | 1.26 |
| Normalized betweenness centrality | 0.56 | 0.00 | 0.04 |
| Normalized characteristic path length | 1.06 | 0.86 | 0.95 |

### *Gothenburg cohort*

|  | **main effect group** | **main effect psychological distress** | **group-by-psychological distress interaction** |
| --- | --- | --- | --- |
| Normalized clustering coefficient | 0.09 | 0.07 | 1.53 |
| Normalized betweenness centrality | 0.01 | 0.72 | 0.42 |
| Normalized characteristic path length | 0.04 | 0.63 | 0.37 |

Values are F-statistics; *p<0.05

## ***3. Reproducibility of graph measures in the pain matrix and DMN network***

## **Methods**

To assess test-retest variability, we calculated graph measures in an independent dataset of healthy subjects scanned twice in exactly the same way as in this study in the same network consisting of the pain matrix and the default mode network. However, the normalization of graph measures was only based on 20 equivalent random graphs.

We used the dataset from the Brain Genomics Superstruct Project (GSP) (Holmes et al., 2015) including 69 healthy subjects (34 males) between 19 and 27 years. They were scanned on a Siemens 3T MAGNETOM Tim Trio MRI system (Erlangen, Germany) using a 12-channel phase-array head coil. Each subject had two sessions of rs-fMRI (eyes closed) within 6 months. 120 images with a TR of 3s were acquired.

Test-retest variability (TRT) (in %) was defined as:

$$\text{TRT}=100\left| \frac{m_{1}-m_{2}}{\frac{m_{1}+m_{2}}{2}} \right|$$

in which m_1_ and m_2_ are the values of the graph measure derived from the first respectively the second measurement. A higher TRT value indicates higher variability and less reproducibility.

The consistency of hubs (HC) is based on the Dice coefficient:

$$\text{HC}=2\frac{\left| \text{H}_{1}\cap\text{H}_{2} \right|}{\left| \text{H}_{1} \right|+\left| \text{H}_{2} \right|}$$

where H_1_ and H_2_ are the list of hubs in the network of the first respectively second measurement. A value of 1 corresponds to a perfect agreement of hubs while 0 reflects no agreement at all.

The reproducibility of the modular structure was assessed by calculating how often (in percentage of subjects) two nodes belong to the same community or module and by comparing this against the probability in equivalent random networks. We performed Fisher exact tests and report results at an uncorrect p < 0.05 and using a FDR corrected p < 0.05.

## **Results**

Test-retest variability of graph measures between the two scans is low (lambda = characteristic path length, C = clustering coefficient, BC = betweenness centrality):

Reproducibility of the identification of hubs:

Reproducibility of the modular structure:

| Node 1 | Node 2 | T1 | T2 |
| --- | --- | --- | --- |
| L Angular | R Angular | **57** | **51** |
| L INS Ant | R INS Ant | **55** | **64** |
| L PCC | R PCC | **52** | **59** |
| L Putamen | R Putamen | **84** | **77** |
| L SI | R SI | **57** | **67** |
| L Thalamus | R Thalamus | **59** | **72** |
| L aMCC | R aMCC | **48** | **49** |
| L dlPFC | R dlPFC | **54** | **62** |
| L pACC | R pACC | **71** | **70** |
| L precuneus | R precuneus | **58** | **70** |
| R Angular | R LTC | **48** | 39 |
| L Amygdala | R Amygdala | 41 | *43* |
| L Hippocampus | R Hippocampus | 39 | **52** |
| L SII | R SII | 29 | *43* |
| L mPFC | R mPFC | 42 | **45** |
| L pMCC | R pMCC | 32 | *43* |

T1 and T2 are the first respectively the second time point. Values which are significant at FDR corrected p < 0.05 are indicated in bold, those significant at uncorrected p < 0.05 are indicated in italic. Pairs in which none of the time points reached significance, are not shown.

# ***4. Comparison of graph measures between HCs from the Sendai and Gothenburg cohort***

## **Methods**

We applied the same methodology as in the main paper, i.e. we calculated graph measures in the network consisting of the pain matrix and the default mode network (DMN), but we limited this analysis to the comparison of the healthy controls between both cohorts and we used only 20 equivalent random graphs to normalize the graph measures.

## **Results**

For the normalized global graph measures, we found significant differences between the healthy controls of both cohorts for the normalized characteristic path length (p = 0.007) and normalized efficiency (p = 0.006). Both survive a Bonferroni correction for the number of global graph measures tested.

# ***5. Comparison with the study of Qi et al.***

## **Methodological differences between Qi et al. and our study**

| **Item** | **Qi et al.** | **Sendai** | **Gothenburg** |
| --- | --- | --- | --- |
| MRI scanner | 3T Siemens | 3T Siemens | 3T Philips |
| fMRI sequence | EPI | EPI | EPI |
| TR (s) | 2.0 | 2.8 | 2.0 |
| # volumes | 250 | 250 | 300 but only first 250 used for graph analysis |
| Voxel size (mm^3^) | 3.75 × 3.75 × 4.0 | 3.4 × 3.4 × 4.0 | 3.4 × 3.4 × 4.0 |
| paradigm | Eyes closed | Eyes closed | Eyes closed |
| Preprocessing | Realignment, slice timing, coregistration, warping | Realignment, slice timing, coregistration, warping | Realignment, slice timing, coregistration, warping |
| Removal of confounding factors | CSF, WM signals  Global signal, motion parameters | CSF, WM signals  Global signal, motion parameters | CSF, WM signals  Global signal, motion parameters |
| Band pass filtering (Hz) | 0.01–0.08 | 0.009 – 0.1 | 0.009 – 0.1 |
| Selection of nodes | Conjunction of correlation based maps using 3 seed regions + selection of regions common for both IBS and HC. The AAL atlas was used to define 20 regions. Unclear if they used the whole parcel from the AAL or only the overlap with the second level conjunction analyses. | Predefined from the most appropriate atlases | Predefined from the most appropriate atlases |
| Functional connectivity | Correlation based on time series within the parcels. Unclear if they used average time series and if they limited this to GM. | Partial correlation based on average time series limited to GM within each region | Partial correlation based on average time series limited to GM within each region |
| Network type | binary | weighted | weighted |
| Density range (%) | 5 - 40 | NA | NA |

## **Re-analysis of our study using the method of Qi et al.**

In order to see if could replicate the finding of Qi *et al.*, we considered the small differences in the acquisition and prepocessing as neglectable. However, the definition of the nodes was clearly different but since we cannot exactly replicate the node definition of Qi *et al.* we decided to keep our definition of the nodes of the default mode network. However, we now used correlations instead of partial correlations and we used binary networks in the same density range as Qi *et al.* to make this part again similar to their approach.

## Results

The main finding in Qi et al. was a significant change between the global efficiency E between HC and IBS patients. Below we show the results for the Gothenburg cohort (left) and the Sendai cohort (right). For this range of densities, we could not find a significant difference (uncorrected p < 0.05) between both groups for any of the cohorts, except for the Gothenburg cohort in which we found a significant difference at one specific density of 19% (p = 0.032). Given the fact that this is found only at one specific density and only with an uncorrected p, we concluded that we were not able to replicate the finding of Qi et al.

Figure caption: Efficiency E as function of density for HC (blue circles) and IBS (red stars).

# **References**

Holmes, A. J., Hollinshead, M. O., O’Keefe, T. M., Petrov, V. I., Fariello, G. R., Wald, L. L., … Buckner, R. L. (2015). Brain Genomics Superstruct Project initial data release with structural, functional, and behavioural measures. Scientific Data, 2, 150031. <https://doi.org/10.1038/sdata.2015.31>

Qi, R. et al. Topological reorganization of the default mode network in irritable bowel syndrome. Molecular neurobiology 53, 6585-6593 (2016).
